# A Bright Spiropyran-Based Zinc Sensor for Live-Cell Imaging

**DOI:** 10.1021/acsomega.5c04186

**Published:** 2025-07-25

**Authors:** Annika M. Pick, Kristin Weber, Marisa F. Jakobs, Max. J. Carlsson, Simon Wittmann, Jörg Fahrer, Sabine Becker

**Affiliations:** † Department of Chemistry, 26562 RPTU Kaiserslautern-Landau, Erwin-Schroedinger-Str. 54, Kaiserslautern 67663, Germany; ‡ Division of Food Chemistry and Toxicology, Department of Chemistry, 26562 RPTU Kaiserslautern-Landau, Erwin-Schroedinger-Str. 52, Kaiserslautern 67663, Germany

## Abstract

Pools of labile bound zinc ions are essential for signal transduction in the human body. At the cellular level, such pools occur in the cytosol, discrete organelles, and secretory vesicles. These zinc-containing vesicles are found in distinct regions of the central nervous system, modulating calcium ion channels that play an essential role in olfaction, audition, and somatosensory perception. Dysregulation of these receptors is associated with a number of neurodegenerative diseases. To understand the underlying mechanisms at the molecular level, zinc fluorescence sensors are versatile tools. In this report, a new member of the spiropyran-based sensor family SpiroZin, which has proven useful for the investigation of zinc in living cells, is presented: SpiroZin2-COOH. This sensor can be synthesized in a 5-step synthesis and shows superior zinc-sensing properties in cuvette as well as live cell studies. The quantum yield is approximately seven times higher than that of the parent zinc sensor, which also results in an approximately 6-fold higher brightness and a turn-on of 30 at pH 7 in cuvette studies. Another advantage is a significant red-shift of 30 nm in comparison to the parent sensor SpiroZin2. Other basic properties of the SpiroZin family are retained, as revealed by a similar binding constant and negligible pH dependence in zinc sensing. Similar to other members of the SpiroZin family, SpiroZin2-COOH images intracellular zinc pools in living cells. Lysotracker costaining reveals lysosomal localization of SpiroZin2-COOH. The turn-on is determined to be 14.6, which is the highest turn-on within the SpiroZin family reported so far in live-cell studies.

## Graphical abstract

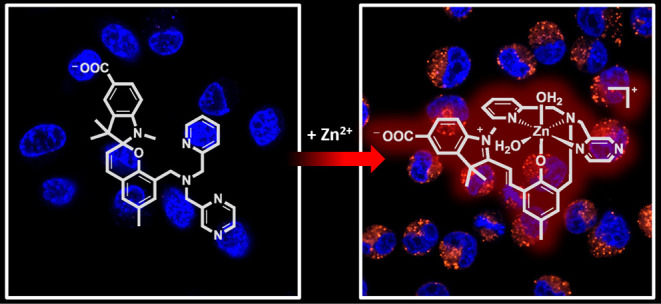

## Introduction

Zinc is an essential nutrient for living organisms.^1^ After iron, it is the second most abundant transition metal, which is highlighted by the high number of approximately 3,000 proteins that depend on zinc.^2,3^ In the human body, zinc fulfills three tasks:^2^ serving as a structural component in a series of proteins, acting as a catalytic cofactor in the active center of enzymes, and functioning as a signaling agent through pools of labile bound zinc ions.^1a,2,4^ These pools occur in the cytosol, discrete organelles, and within secretory vesicles.^5^ Emerging evidence points to a crucial role of such pools of zinc ions in olfaction^6a,6i^ audition,^6b,6i^ and somatosensory perception.^6^ In the brain, such zinc-containing vesicles are found at the presynaptic terminal of glutamatergic neurons, which are predominant in the hippocampus, amygdala, dorsal cochlear nucleus, and cortex.^7^ During synaptic transmission, zinc is coreleased with the neurotransmitter glutamate and inhibits ion channels on the postsynaptic side, such as the α-amino-3-hydroxy-5-methyl-4-isoxazolepropionic acid receptor (AMPAR) and *N*-methyl-*D*-aspartate receptor (NMDAR).^6c–6f,8^ In this context, both, the modulation by secretory-released zinc and tonic zinc levels are important for a normal receptor function.^9^ Owing to its crucial role in the CNS, an abnormal function of the NMDAR is associated with a number of neurodegenerative and mental health disorders such as Alzheimer’s, Huntington’s, and Parkinson’s disease, as well as schizophrenia, stroke, autism, and diverse mood disorders such as depression.^10^ Even though the profound knowledge of zinc signaling at the molecular level seems to be essential for the comprehension and possible treatment of such diseases, the underlying processes are only poorly understood.

Fluorescent zinc sensors have been proven to be useful tools for zinc imaging in live cells, and accordingly, several different classes have been developed.^11^ Among these, spiropyran-based sensors stand out for their high pH stability, which is ensured by a reaction-based, rather than photoinduced electron transfer (PET)-based sensing mechanism. This relative pH independence makes spiropyran-based sensors superior for probing secretory vesicles that usually are more acidic than the cytosol or extracellular space;^12^ e.g., a recent study highlights the superiority of SpiroZin2 over FluoZin-3, a fluorescein-based sensor, in sensing zinc in secretory vesicles.^13^ Zinc binding induces a change from the nonfluorescent spiroform (SP) to the fluorescent merocyanine form (MC) that coordinates Zn^2+^ (Scheme 1). To date, spiropyran derivatives that are equipped with diverse chelating units are used for zinc and other metal ion detection.^14^ In 2014 and 2015, Lippard et al. introduced SpiroZin1 and 2, which are equipped with a dipicolylamine and a pyrazin-2-ylmethyl-pyridin-2-ylmethyl-amine chelating site, respectively (Scheme 1)^14c,14d^ that can be used for zinc imaging in living cells.

**Scheme 1:**
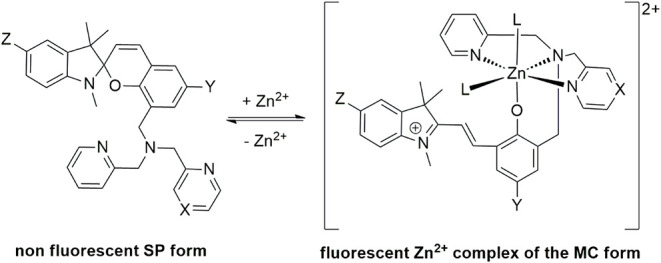
Zinc Sensing Mechanism of SpiroZin Sensors (L = H_2_O and/or Anion^15^ ^a^

a SpiroZin1 (*X* = C, *Y* = CH_3_, *Z* = H), SpiroZin2 (*X* = N, *Y* = CH_3_, *Z* = H), and further analogues of spiropyran by Heng et al. (*X* = C, *Y* = NO_2_/F, *Z* = COOH)

In 2017, Heng et al. reported analogues of SpiroZin1, in which a carboxylic group was introduced into the indole moiety. Additionally, the methyl group in the aromatic sensor backbone was replaced by a 6’-fluoro substituent or an NO_2_-substituent (Scheme 1). The 6’-fluoro analogue allowed for monitoring the Zn^2+^ efflux from cells that underwent apoptosis;^14e^ however, both analogues displayed a smaller turn-on upon zinc addition than SpiroZin1 and 2.^14c–14e^

An advantage of these analogues, however, is the improved hydrophilicity due to the introduction of the carboxylic group. The resulting water solubility allows for avoiding DMSO as a solvent, which has been shown to be potentially harmful in cell culture and can lead to side effects on gene expression as well as cross-organ interactions.^16^

In this report, we present the spiropyran-based sensor SpiroZin2-COOH that combines the advantages of previously known sensors with water solubility, an advantageous red-shifted emission, an extraordinary turn-on, and a comparatively high quantum yield, allowing for the sensitive detection of intracellular zinc by means of live-cell imaging.

## Results and Discussion

SpiroZin2-COOH was obtained via a 5-step synthesis (Scheme 2). The reductive amination of pyridine-2-carbaldehyde and C-pyrazine-2-yl-methylamine led to pyrazin-2-ylmethyl-pyridin-2-ylmethyl-amine (**1**). 2-Hydroxy-5-methyl-benzaldehyde was converted to 3-bromomethyl-2-hydroxy-5-methyl-benzaldehyde (**2**) in a one-step synthesis. Through a Fischer-indole synthesis and subsequent methylation with MeI, 4-hydrazino-benzoic acid yielded 5-carboxy-1,2,3,3-tetramethyl-3H-indolium (**4**). Similar to the synthesis reported for SpiroZin2, **1**, **2**, and **4** were allowed to react in a one-pot reaction to yield SpiroZin2-COOH.^14d^

**Scheme 2:**
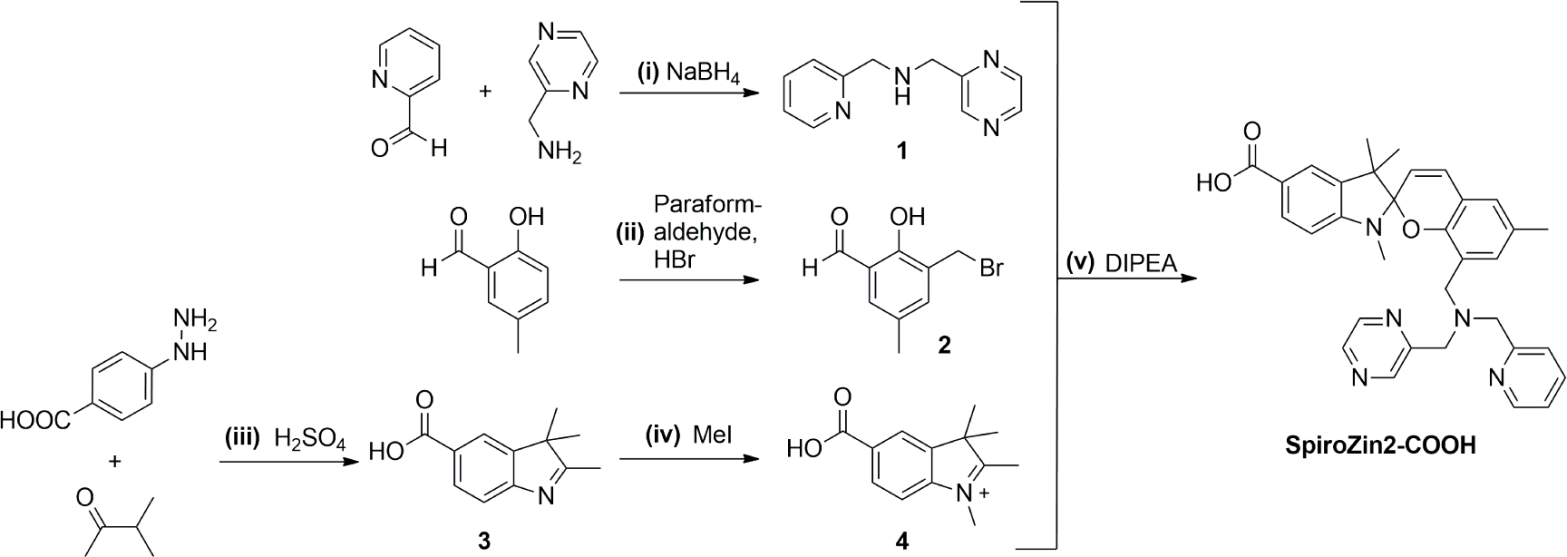
Synthetic Route to SpiroZin2-COOH

SpiroZin2-COOH was obtained as a red solid after MPLC purification, followed by HPLC purification (SI Chapter 1.5). It forms an orange solution in DMSO and water. Upon addition of ZnSO_4_·7H_2_O, the color of this solution changes to bright pink (Figure S23). This color change is reflected in the absorption spectra of the zinc-free and zinc-bound forms in PIPES buffer. The spectrum of pure SpiroZin2-COOH shows maxima at 267 and 294 nm, deriving from aromatic π–π* transitions (Figure 1), and accordingly resembles those of known spiropyran-based zinc sensors.^14c–14e^

**Figure 1:**
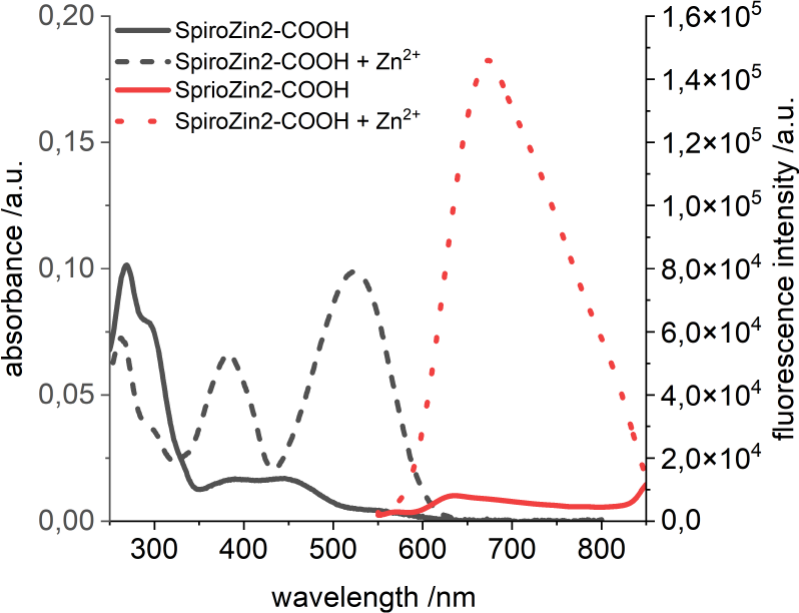
Absorption (black lines) and fluorescence (red lines) spectra of 5 μM SpiroZin2-COOH in aqueous buffer (50 mM PIPES, 100 mM KCl, pH 7) before (solid lines) and after (dotted lines) the addition of 100 equiv of ZnSO_4_. Photophysical properties: λ_abs_ = 526 nm (ε_526_ = 2,6(8) × 10^4^ cm^–1^ M^–1^); λ_em_ = 675 nm (ϕ = 0.0065(6)).

Upon zinc addition, the π–π* absorption band at 267 nm is shifted hypsochromically to 261 nm, and two new maxima at 383 and 526 nm are formed, which are characteristic of the MC zinc complex.^14c–14e^ In comparison to SpiroZin2, the main absorption maximum at 526 nm is bathochromically shifted by 8 nm. The extinction coefficient (λ_abs_ = 526 nm) was determined to be ε_526_ = 2.6(8) × 10^4^ cm^–1^ M^–1^, which is in good agreement with the one reported for SpiroZin2 (Table 1).^14d^

**Table 1:** Photophysical Properties of Spiropyran-Based Zinc sensors^a^ ^b^ ^c^ ^d^ ^e^

	SpiroZin1[^a^]	SpiroZin2[^a^]	spiropyran-F [^b^]	spiropyran-NO_2_[^b^]	SpiroZin2-COOH[^a^]
λ_abs_/nm	508	518	appr. 514	appr. 514	526
ε/cm^–1^ M^–1^	1.40(3) × 10^4^	3.071(1) × 10^4^	n.d.	n.d.	2.6(8) × 10^4^
λ_em_/nm	650	645	670	615	675
ϕ	0.0042(7)	0.0010(1)	0.0028	0.0030	0.0065(6)^[c]^
brightness^[d]^/10^4^	0.006	0.003	n.d.	n.d.	0.0169
turn-on	6–7	n.d.	4	1.5	30
turn-on (live-cell studies)	n.d.	12^[e]^	n.d.	n.d.	14.6
*K*_d_	21(1) pM	3.6 nM	n.d.	n.d.	3.1 nM

a Determined in aqueous buffer (50 mm PIPES, 100 mm KCl, pH 7).

b Determined in Water.

c For zinc-containing forms (see SI, Chapter 2.9).

d Defined as the product of quantum yield and extinction coefficient (Φ × ε).

e Determined in living cells,

In the zinc-free form, SpiroZin2-COOH does not show any fluorescence in the range of 550–800 nm, indicating that the molecule is in the nonfluorescent SP form (Figure 1). The addition of zinc leads to deep red fluorescence between 600 and 850 nm. In comparison to SpiroZin1 and 2, the emission band is broader with an asymmetrical form; however, it corresponds well with that observed for carboxylated spiropyran derivatives.^14e^ Compared to the known four spiropyran-based sensors, SpiroZin2-COOH exhibits, with 675 nm, the most red-shifted maximum (Table 1). The quantum yield of zinc-bound SpiroZin2-COOH was determined against tetraphenylporphyrin (Figure S31) to ϕ = 0.0065 (6) and is approximately seven times higher than that of the parent zinc sensor SpiroZin2 (Table 1). As a consequence, SpiroZin2-COOH is also approximately 6-fold as bright as SpiroZin2. This considerably high quantum yield and brightness represent a significant advantage for zinc imaging in living cells. It is also in accordance with the higher turn-on of SpiroZin2-COOH in comparison to known spiropyran-based sensors.

This turn-on is determined to be 30 (pH 7, integration range 675 to 725 nm), which is also significantly higher than those reported for known spiropyran-based sensors. Among those sensors, SpiroZin1 yielded the highest turn-on reported so far, 6–7 (Table 1).^14c^ The turn-on of the parent sensor SpiroZin2 was not reported for cuvette studies; however, in live-cell studies, SpiroZin2 yields a turn-on of approximately 12 (Table 1).^14d^ The dissociation constant *K*_d_ was determined to be 3.1 nM and, thus, is comparable to that of unsubstituted SpiroZin2 (Table 1).

In pH-dependent studies, SpiroZin2-COOH retained its high turn-on throughout a pH range of 7 to 10 with a maximum turn-on of 30 at pH 7. Neither the low background fluorescence nor the turn-on upon zinc addition responds decisively to changes in pH values at basic levels (Figure S29). At pH 5 and 6, the lowest response to zinc (turn-on: 10.4 and 9.9, respectively) was observed; however, the turn-on increased under more acidic conditions and reached 21.41 at pH 3. Similar to SpiroZin1 and 2, SpiroZin2-COOH thus proves to be a suitable zinc sensor for application at various pH values. As also observed for SpiroZin2, SpiroZin2-COOH shows the highest turn-on at pH 7 (and pH 8). These similarities show that the additional carboxylic group of SpiroZin2-COOH does not alter the pH dependency of the turn-on upon zinc addition.

The selectivity for zinc ions was assessed in the presence of other metal ions. Therefore, a solution of the competing metal salt of interest was added to a SpiroZin2-COOH solution. After the fluorescence intensity of the resulting mixture had been assessed, a solution of ZnSO_4_ was added and another fluorescence spectrum was recorded. These studies revealed a highly selective response toward zinc ions as the addition of Na^+^, Mg^2+^, Ca^2+^, Mn^2+^, Fe^3+^, Ni^2+^, and Cu^2+^ alone did not lead to a notable turn-on (Figure 2). Subsequent addition of Zn^2+^ led to a strong increase in the fluorescence intensity for solutions containing Na^+^, Mg^2+^, Ca^2+^, Mn^2+^, and Fe^3+^. Except for the solution containing Mn^2+^, fluorescence levels close to those of pure SpiroZin2-COOH were reached (≥90%). For Mn^2+^, only approximately 60% of the fluorescence intensity for SpiroZin2-COOH was observed. This behavior is in accordance with that of SpiroZin2, for which a complete restoration of fluorescence in the presence of Mn^2+^ also was not observed.^14d^ Notably, when using SpiroZin1, the addition of Mn^2+^ led to a complete quenching of fluorescence, even after subsequent addition of Zn^2+^.^14c^

**Figure 2:**
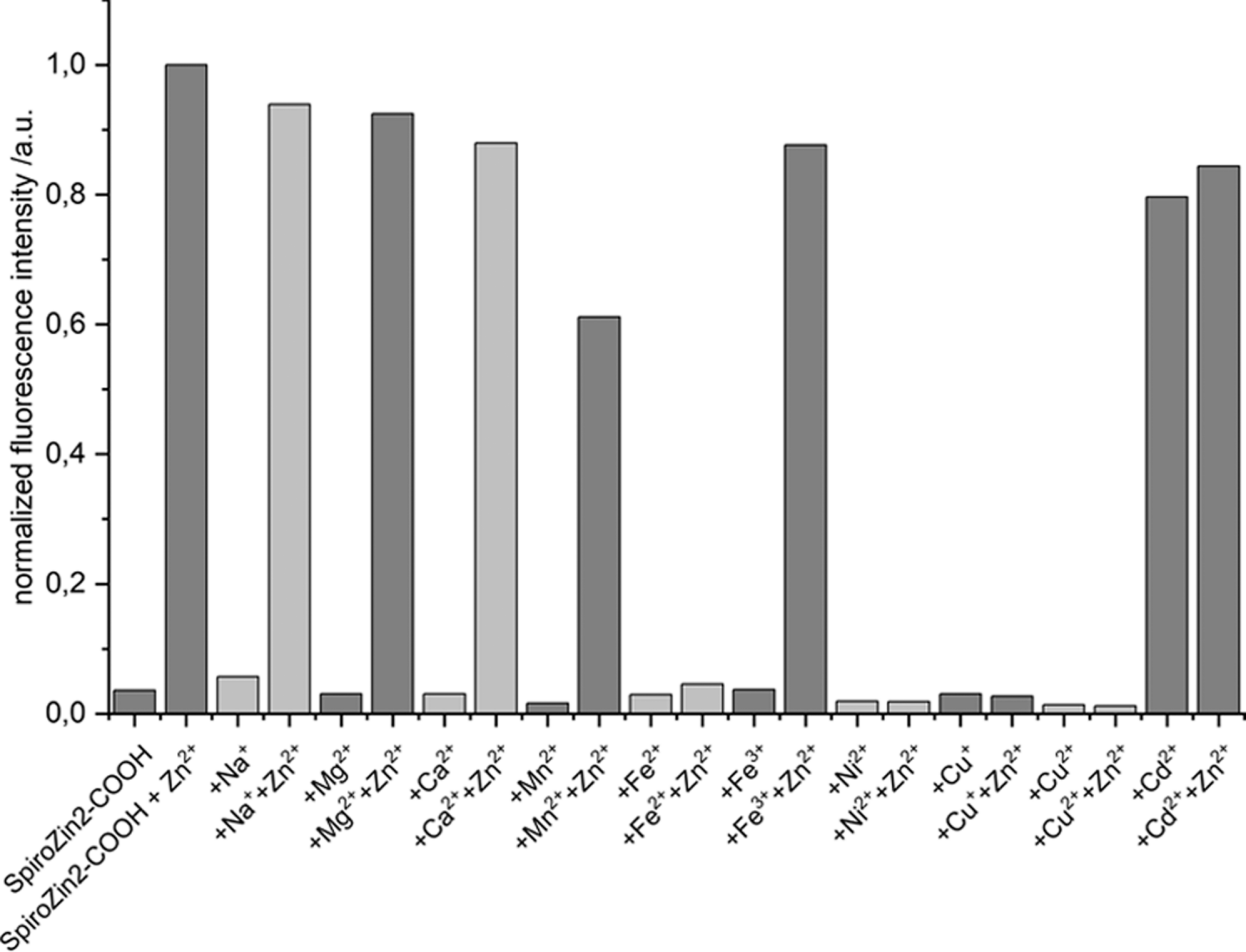
Influence of chosen metal ions on the fluorescence intensity of SpiroZin2-COOH. Intensities are normalized to that of the SpiroZin2-COOH zinc complex.

In contrast, the addition of Cd^2+^ to the solution of SpiroZin2-COOH did lead to a similar turn-on as the addition of Zn^2+^. Consequently, further addition of Zn^2+^ did not lead to a significant increase in the fluorescence intensity. This simultaneous response to Cd^2+^ is known for most zinc sensors. Accordingly, known spiropyran-based sensors also fail to discriminate between Zn^2+^ and its heavier analogue Cd^2+^. Also, in accordance with known spiropyran-based sensors, the addition of the open-shell metal ions Fe^3+^, Cu^2+^, and Ni^2+^ did not lead to any increase in fluorescence, neither before nor after the addition of Zn^2+^ as they quench the fluorescence. The same observation was made for Fe^2+^ and Cu^+^.

Having a functional zinc sensor at hand, the ability of SpiroZin2-COOH to detect zinc in living cells was investigated using confocal microscopy. To compare the results to those from previous studies, HeLa cells were used. The cells were incubated with a 10 μM solution of SpiroZin2-COOH and Hoechst 33342 to stain nuclei (20 μM, Figure 3A). Prior to the addition of zinc pyrithione, red fluorescence was not observed, indicating that SpiroZin2-COOH was still in the nonfluorescent SP form. Adding 10 equiv of zinc pyrithione (100 μM) led to red fluorescence (Figure 3A). Comparison of the signal in the red channel before and after the addition of zinc pyrithione revealed a turn-on of 14.6 (Figure 3B). To confirm that the turn-on derived from zinc complexation, the chelator N,N,N′,N′-tetrakis­(2-pyridinylmethyl)-1,2-ethanediamine (TPEN) was added in 20-fold excess. This treatment resulted in a turn-off of the red fluorescence signal (Figure 3B); however, in comparison to the initial signal prior to zinc addition, a small fluorescence signal was still observed, indicating that not all zinc ions have been removed from the sensor by TPEN.

**Figure 3:**
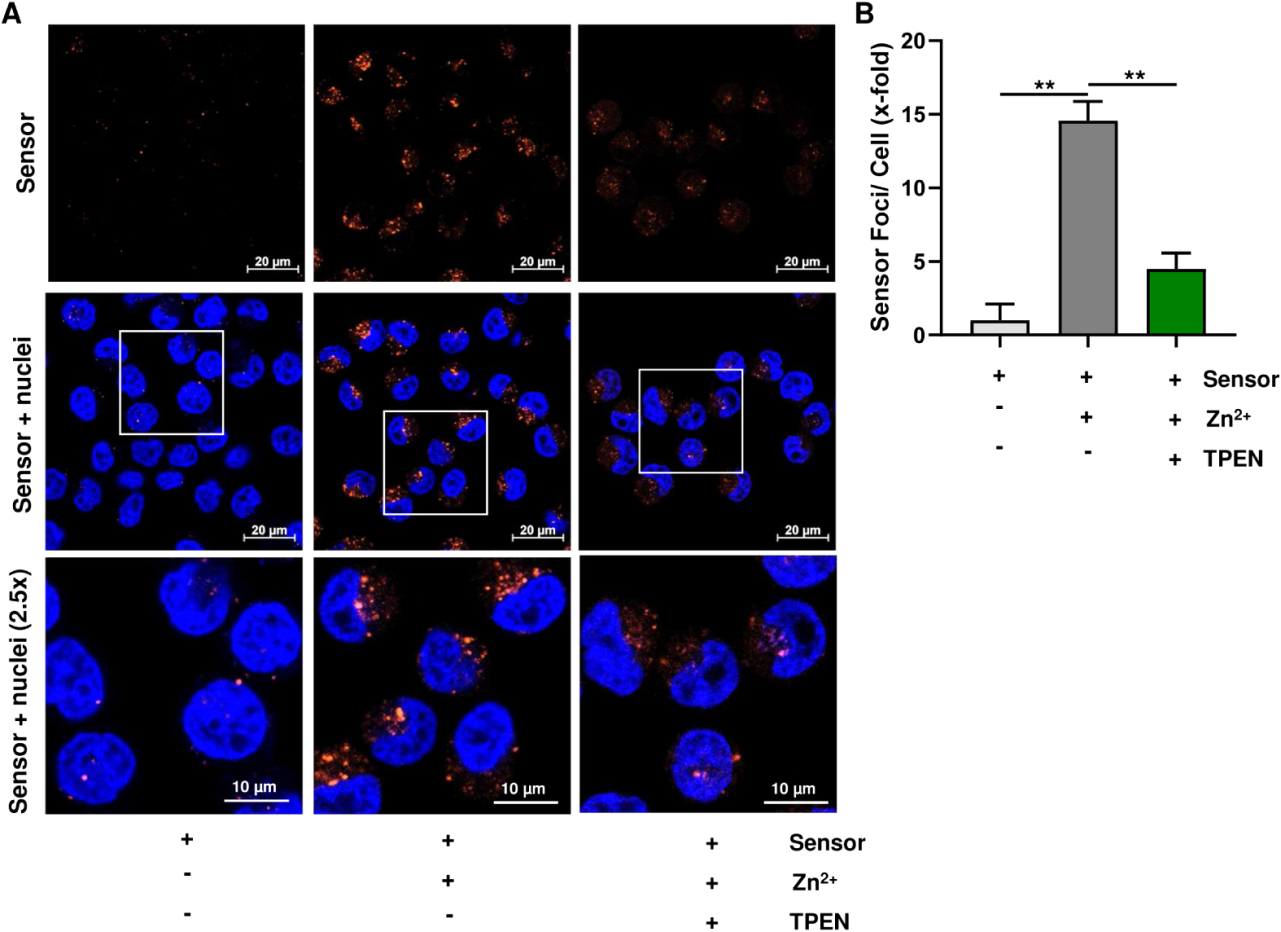
A. Confocal fluorescence microscopy of living HeLa cells pretreated with 5 μM SpiroZin2-COOH before and after subsequent addition of 50 μM zinc pyrithione in water (10 equiv) and addition of 50 μM TPEN (20 equiv). Nuclei were counterstained with Hoechst 33342. Representative images are depicted (*n* = 3). B. Fluorescence normalized to the background fluorescence of the cells after the addition of SpiroZin2-COOH. Data are shown as mean + SEM (*n* = 3). **p < 0.01.

The subcellular localization of SpiroZin2-COOH was examined using LysoTracker Green DND-26, which localizes to lysosomal acidic vesicles (Figure 4A). Therefore, the cells were coincubated with Hoechst 33342, SpiroZin2-COOH (5 μM), zinc pyrithione (50 μM), and LysoTracker Green DND-26 (33 nM). The Pearson’s correlation coefficient was determined to be 0.80, showing the colocalization of SpiroZin2-COOH and lysosomes (Figure 4B), thus resembling the behavior of SpiroZin1 and 2.

**Figure 4:**
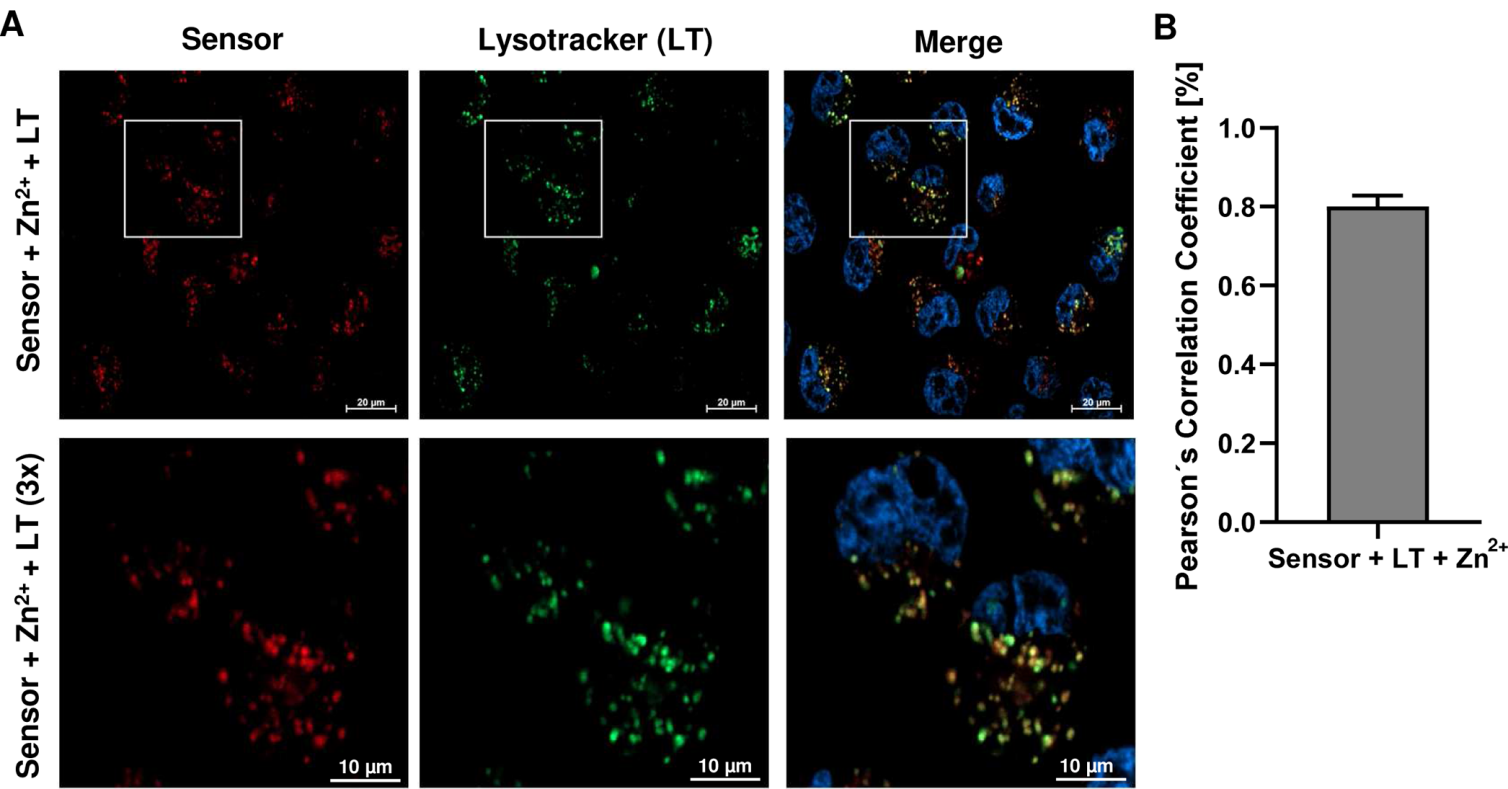
A. Confocal fluorescence microscopy images of living HeLa cells pretreated with 20 μM Hoechst 33342, 5 μM SpiroZin2-COOH, 50 μM zinc pyrithione, and 33 nM LysoTracker Green DND-26. Representative images are depicted (*n* = 3). B. Determination of Pearson’s correlation coefficient. Data are shown as mean + SEM (*n* = 3).

## Conclusions

Simple derivatization of existing sensors can lead to superior properties, as shown in this case study. We introduced a carboxylic group into the sensor design of the versatile zinc fluorescent sensor SpiroZin2. The resulting sensor SpiroZin2-COOH not only shows improved water solubility but also shows improved zinc sensing properties in cuvettes as well as in live-cell studies. Compared to SpiroZin2, SpiroZin2-COOH has an advantageous red-shifted absorption (by 8 nm) and emission (by 30 nm), a quantum yield approximately seven times higher and a slightly higher turn-on in live-cell studies. At the same time, the binding constants remain similar. Also, the incorporation of the carboxylic group did not change the localization in living HeLa cells. In conclusion, SpiroZin2-COOH represents a novel fluorescent zinc sensor with highly improved sensing properties that can be proved useful for the investigation of zinc in living cells and tissue studies.

## Experimental Section

### General Materials and Methods

All chemicals used were of p.a. quality and purchased from ABCR, Acros Organics, Alfa Aesar, Carbolution, Merck, Roth, TCI, or Sigma-Aldrich. Following the literature, some compounds were synthesized under a nitrogen atmosphere. Standard Schlenk techniques were used. Synthesis and additional analytical data are given in the Supporting Information.

### Photophysical and Zinc-Binding Properties of Spirozin2-COOH

If not otherwise specified, all spectroscopic measurements were performed in an aqueous buffer (PIPES, pH 7.0). Fluorescence measurements were obtained by excitation at 518 nm, acquisition from 500 to 900 nm, and a slit width of 20 nm. The quantum yield was standardized to TPP (tetraphenylporphyrin, Φ = 0.11 at λ_ex_ = 490 – 610 nm) in toluene with an excitation wavelength of 550 nm.^17^

### General Materials and Methods for Live-Cell Imaging

HeLa cells were cultivated in 75 cm^2^ cell culture flasks at 37 °C, 5% CO_2_, and 97% relative humidity. The cells were cultured in DMEM (1×) (Dulbecco’s modified Eagle medium; Gibco Life Technologies, Germany) supplemented with 10% FBS (fetal bovine serum; PAN-Biotech, Germany) and antibiotics (100 U/mL penicillin and 100 μg/mL streptomycin). For the incubation procedures, DMEM was used without additives. Cell imaging was performed using a confocal laser scanning microscope (LSM900, Zeiss, Oberkochen, Germany), as reported previously.^18^

## Supplementary Material



## Notes

NMR (^1^H and ^13^C), IR, ESI-mass, UV/vis, and fluorescence spectra; information on the determination of (photophysical) characteristics; and cell studies (including preparation) (PDF)
