# Supplementary material for: A Bright Spiropyran-Based Zinc Sensor for Live-Cell Imaging
Source: ACS Omega. 2025 Jul 25;10(30):33629–35. doi: 10.1021/acsomega.5c04186 (PMC12332670; doi:10.1021/acsomega.5c04186)
Supplement: Supplementary file 1 [file ao5c04186_si_001.pdf]

# A Bright Spiropyran-Based Zinc Sensor for Live Cell Imaging

Annika M. Pick, Kristin Weber, Marisa F. Jakobs, Max Carlsson, Simon Wittmann, Jörg Fahrer, and Sabine Becker*

[a] A. M. Pick, K. Weber, M. F. Jakobs, S. Becker*
Department of Chemistry
RPTU Kaiserslautern-Landau
Erwin-Schroedinger-Str. 54, 67663 Kaiserslautern

[b] M. Carlsson, S. Wittmann, J. Fahrer
Division of Food Chemistry and Toxicology
Department of Chemistry
RPTU Kaiserslautern-Landau
Erwin-Schroedinger-Str. 52, 67663 Kaiserslautern

*zinc • zinc sensor • fluorescence • live cell imaging • spiropyran*

# Table of Contents

1. Experimental Procedures ........ 3

**1.1 Synthetic Protocols and Procedures** ........ 3

**1.2 NMR Spectroscopy** ........ 5

**1.3 IR Spectroscopy** ........ 10

**1.4 Mass Spectrometry** ........ 13

**1.5 Chromatographic purification methods** ........ 15

2. Photophysical Properties of SpiroZin2-COOH ........ 16

**2.1 UV/vis Spectroscopy** ........ 16

**2.2 Absorption Spectra** ........ 16

**2.3 Extinction Coefficient** ........ 17

**2.4 Fluorescence Spectroscopy** ........ 18

**2.5 Emission Spectra** ........ 18

**2.6 Affinity Studies** ........ 19

**2.8 pH Dependency** ........ 20

3. Cell Culture and Live Cell Imaging ........ 23

References ........ 24

# 1. Experimental Procedures

**General Materials and Methods**

All chemicals used were of p.a. quality and purchased from ABCR, Acros Organics, Alfar Aesar, Carbolution, Merck, Roth, TCI or Sigma Aldrich. Some compounds were synthesized under nitrogen atmosphere. Standard Schlenk techniques were used for this procedure.

**1.1 Synthetic Protocols and Procedures**

**Pyrazin-2-ylmethyl-pyridin-2-ylmethyl-amine** (**1**): Synthesis was carried out similarly to the published procedure by *Lippard et al*.[1]

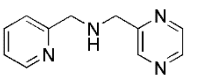


Pyridine-2-carbaldehyde (0.50 g, 0.44 mL, 4.7 mmol, 1 eq.) was dissolved in methanol (45 mL) and pyrazine-2-ylmethane-amine (0.50 g, 0.35 mL, 4.7 mmol, 1 eq.) was added. The reaction solution was stirred for 30 min. Then, $NaBH_4$ (0.53 g, 14 mmol, 3 eq.) was added via small portions. The resulting solution was stirred for another 2 h. The solvent was removed and the residue re-dissolved in water (20 mL). The product was extracted with dichloromethane (3 x 30 mL). The organic phases were combined and the solvent was removed. The product was purified via MPLC (medium pressure liquid chromatography using method 1 (Table S1).

Yield: 69 % (0.64 g, 3.2 mmol) of a yellow oil.

$^1$H-NMR (400 MHz, $CDCl_3$): δ/ppm = 8.64 (s, 1H), 8.56 (d, $^3J$ = 8 Hz, 1H), 8.52 (d, $^3J$ = 4 Hz, 1H), 8.45 (d, 1H), 7.63 (t, $^3J$ = 8 Hz, 1H), 7.32 (d, $^3J$ = 12 Hz, 1H), 7.16 (t, $^3J$ = 12 Hz, 1H), 4.02 (s, 2H), 3.98 (s, 2H).

$^{13}$C-NMR (400 MHz, $CDCl_3$) δ/ppm = 159.34, 155.36, 149.53, 144.46, 144.18, 143.27, 136.67, 122.46, 122.23, 54.85, 52.45.

IR (KBR, cm$^{-1}$): 3402.8 (N-H), 3314.1, 3053.7 (C-H, aromatic), 3010.3, 2918.7 (C-H, aliphatic), 2834.8 (C-H, aliphatic), 1591.1 (δ-N-H), 1526.4 (C-C, aromatic), 1474.3 (C-C, aromatic), 1403.0, 1303.6, 1238.1, 1149.4, 1054.9, 1018.2, 827.3 (2 adjecent H, aromatic), 788.9 (4 adjecent H, aromatic), 629.6.

ESI-MS (*m*/*z*, **positive mode, sprayed in acetonitrile**): 201.13 $[M+H]^+$, 107.13 [frag, $C_6H_7N_2]^+$.

Retention time: 40.98 min (MPLC, PF-30SIHP-F0040, 60 % ethyl acetate, 40 % ethanol).

**3-bromomethyl-2-hydroxy-5-methyl-benzaldehyde** (**2**): Synthesis was carried out under inert conditions ($N_2$ atmosphere, dry

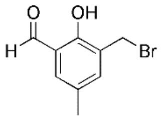


solvents) on the basis of a previously described procedure.[2] 5-methyl-2-hydroxybenzaldehyde (4.15 g, 30.5 mmol, 1 eq.) and paraformaldehyde (1.35 g, 45.4 mmol, 1.5 eq.) were suspended in HBr (20 mL) and heated to 70 °C. The reaction mixture was stirred for 48 h and then cooled to room temperature. A portion of HBr (10 mL) was added to the mixture, which then was heated to 80 °C and stirred for another 19 h. The reaction mixture was allowed to cool to room temperature and was quenched with water (20 mL). The product was extracted with dichloromethane (40 mL, 20 mL, 10 mL). The organic phases were combined and dried over $Na_2SO_4$. The drying agent was filtered off and the solvent was removed to yield **2**.

Yield: 94 % (6.58 g, 28.7 mmol) colorless solid.

$^1$H-NMR (400 MHz, $CDCl_3$): δ/ppm = 11.30 (s, 1 H), 9.86 (s, 1 H), 7.43 (d, $^4J$ = 1.6 Hz, 1 H), 7.33 (d, $^4J$ = 1.6 Hz, 1 H), 4.56 (s, 2 H), 2.34 (s, 3 H).

$^{13}$C-NMR (105 MHz, $CDCl_3$): δ/ppm = 20.32, 26.81, 120.64, 126.17, 129.41, 134.31, 139.01, 157.55, 196.53.

IR (ATR, cm$^{-1}$): 3178.11 (O-H), 3039.26 (C-H, aromatic), 2852.20 (C-H, aliphatic), 1658.48 (C=O), 1595.81, 1467.56 (*δ*-$CH_2$), 1419.35 (*δ*-OH), 1376.93 (sym. *δ*-$CH_3$), 1294.00, 1267.97, 1208.18, 1163.83, 1121.40, 976.76, 873,60 (C-H, aromatic), 698.11 (C-Br), 591.08.

ESI-MS (*m*/*z*, positive mode, sprayed in acetonitrile): 282.25, 249.10 $[M-Br+COOH+Na+MeOH]^+$, 217.10 $[M-Br+COOH+Na]^+$, 195.32 $[M-Br+HCOOH]^+$, 163.15 $[M-H_2O-Br+MeOH]^+$.

**2,3,3-Trimethyl-3H-indole-5-carboxylic acid** (**3**): Synthesis was carried out under inert conditions ($N_2$ atmosphere, dry solvents)

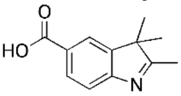


following the procedure described by *Tomasulo et al*.[3] 4-hydrazinobenzoic acid (1.00 g, 6.57 mmol, 1 eq.) was dissolved in ethanol (30 mL). 3-methyl-2-butanone (0.8 mL, 0.64 g, 7.5 mmol, 1.1 eq.) and concentrated $H_2SO_4$ (0.2 mL) were added. The reaction mixture was heated to 90 °C and stirred at this temperature for 16.5 h. After cooling to room temperature, the solvent was removed. The red, crude solid was purified via MPLC (medium pressure liquid chromatography) using method 2 (Table S3).

Yield: 61 % (813 mg, 3.99 mmol) orange colored solid.

$^{1}$H-NMR (400 MHz, $CDCl_3$): δ/ppm = 8.15 (d, $^{3}J$ = 8 Hz, 1 H), 8.06 (s, 1 H), 7.68 (d, $^{3}J$ = 8 Hz, 1 H), 2.39 (s, 3 H), 1.37 (s, 6 H).

$^{13}$C-NMR (105 MHz, $CDCl_3$): δ/ppm = 192.57, 171.31, 157.93, 145.82, 131.02, 126.67, 123.39, 119.86, 54.06, 23.02, 15.78.

IR (KBr, cm$^{-1}$): 3052.76, 2967.91, 2931.27, 2866.67, 2825.20, 2575.47, 2539.79, 1908.22, 1685.48, 1617.02, 1582.31, 1421.28, 1384.64, 1343.18, 1297.86, 1233.25, 1152.26, 1112.73, 996.05, 945.91, 904.45, 855.28, 759.82.

Retention time: 54.72 min (MPLC, PF-50SIHP-F0120, 38 % pentane, 62 % ethyl acetate).

**5-carboxy-1,2,3,3-tetramethyl-3H-indolium iodide** (**4**): Synthesis was carried out under inert conditions ($N_2$ atmosphere, dry solvents) following the procedure described by *Tomasulo et al*.[3] Compound **3** (166 mg, 0.817 mmol, 1 eq.) and methyliodide (0.05 mL, 114 mg, 0.803 mmol, 1 eq. ) were dissolved in absolute toluene (10 mL) and acetonitrile (7 mL). The reaction mixture was heated to 105 °C and stirred under reflux at this temperature for 15 h. Then, the reaction mixture was cooled to 0 °C. The pale pink colored precipitate was filtered off and washed with a small portion of ethanol (20 mL) and hexane (2 x 20 mL).

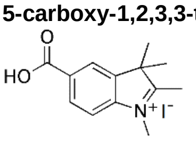


Yield: 48 % (137 mg, 0.628 mmol) of a pale pink solid.

Main product (a):
$^{1}$H-NMR (400 MHz, DMSO): δ/ppm = 8.37 (s, 1 H), 8.18 (d, $^{3}J$ = 8 Hz, 1 H,), 8.018 (d, $^{3}J$ = 8 Hz, 1 H), 3.99 (s, 3 H), 2.81 (s, 3 H), 1.57 (s, 6 H).

$^{13}$C-NMR (105 MHz, DMSO): δ/ppm = 199.07, 166.52, 145.29, 141.97, 131.61, 130.40, 124.22, 115.41, 54.28, 35.05, 21.51, 14.64.

IR (ATR, cm$^{-1}$): 3444.24 (*v*-O-H), 3062.41 (*v*-C-H, aromatic), 2978.52 (*v*-C-H, aliphatic), 2707.6, 2642.00, 2588.00, 2537.9, 1726.94, 1691.27 (C=O), 1619.91, 1594.84 (C=C, aromatic), 1470.46, 1433.82 (*δ* -$CH_2$), 1351.86, 1302.68, 1187.94, 1253.5, 1164.79, 111.8, 770.42 (*δ* -C-H, aromatic), 712.57.

By-product (b):

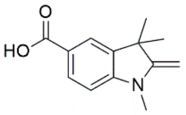


$^{1}$H-NMR (400 MHz, DMSO): δ/ppm = 1.29 (d, $^{3}J$ = 4 Hz, 6 H), 3.06 (s, 1 H), 4.01 (d, $^{3}J$ = 4 Hz, 3 H), 6.73 (d, $^{3}J$ = 8 Hz, 1 H), 7.66 (s, 1 H), 7.75 (d, $^{3}J$ = 8 Hz, 1 H).

**SpiroZin2-COOH**: Synthesis was carried out under inert conditions ($N_2$ atmosphere, dry solvents) and analogously to that of SpiroZin2.[1] Compound **1** (200 mg, 1.0 mmol, 1 eq.) and **2** (229 mg, 1.0 mmol, 1 eq.), were dissolved in ethanol. DIPEA (0.52 mL, 387 mg, 3.0 mmol, 3 eq.) was added to the stirred solution and the reaction mixture was stirred for 1 h at 70 °C. **4** (393 mg; 1.14 mmol, 1.14 eq.) was added and the reaction mixture was stirred for another 2 h at 90 °C under reflux. The solvent was removed and the crude product was purified via HPLC (see Table S3 and Table S4)

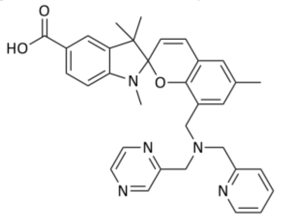


Yield of crude product: 1.27 g of brown-orange colored solid.

$^{1}$H-NMR (400 MHz, $CD_3OD$): δ/ppm = 1.19 (s, 3 H), 1.32 (s, 3 H), 2.22 (s, 3 H), 2.89 (s, 3 H), 4,08−4.18 (m, 1 H), 4.34 (s, 4 H), 4.37−4.41 (m, 1 H), 5.85 (d, $^{3}J$ = 12 Hz, 1 H), 6.52 (d, $^{3}J$ = 8 Hz, 1 H), 6.97−7.02 (m, 1 H), 7.31 (d, $^{3}J$ = 8 Hz, 1 H), 7.45 (td, $^{3}J_t$ = 8 Hz, $^{4}J_d$ = 4 Hz, 3 H), 7.69 (d, $^{3}J$ = 4 Hz, 1 H), 7.81 (dd, $^{3}J_d$ < 4 Hz, $^{4}J_d$ < 4 Hz, 1 H), 7.90 (dt, $^{3}J_t$ = 8 Hz, $^{4}J_d$ = 4 Hz, 1 H), 8.44 (d, $^{3}J$ = 4 Hz, 1 H), 8,46 (t, $^{3}J$ = 4 Hz, 1 H), 8.50−8.53 (m, 1 H).

$^{13}$C-NMR (105 MHz, $CD_3OD$): δ/ppm = 20.32, 20.40, 26.28, 29.08, 52.58, 55.36, 56.95, 58.35, 106.93, 107.14, 117.76, 120.60, 120.95, 122.35, 124.46, 125.46, 125.71, 130.58, 130.96, 131.78, 132.59, 134.16, 137.83, 140.85, 145.41, 145.88, 146.09, 148.68, 149.51, 151.89, 152.27, 153.05, 170.26.

IR (KBr, cm-1): 3444 (v-O-H), 3062 (v-C-H, aromatic), 2979 (v-C-H, aliphatic), 2708, 2642, 2588, 2538, 1691 (v-C=O), 1595, 1470, 1434 (δ -CH2), 1353, 1303, 1254, 1165, 1112, 1058, 945, 845, 770 (δ -C-H, aromatic), 712.

ESI-MS (*m*/*z*, positive mode, sprayed in acetonitrile): 548.25 $[M+H]^+$

Retention time: 10.79 min (HPLC, US5PHC4-250/46, 40 % $H_2O$, 60 % $CH_3CN$, 0.1 % TFA) & 13.64 (HPLC, US5PHC4-250/46, 27 % $H_2O$, 73 % $CH_3CN$, 0.1 % TFA).

Retention time: 18.04 min (HPLC, US5PHC4-250/100, 44 % $H_2O$, 56 % $CH_3CN$, 0.1 % TFA) & 22.55 (HPLC, US5PHC4-250/100, 26 % $H_2O$, 74 % $CH_3CN$, 0.1 % TFA).

**1.2 NMR Spectroscopy**

The nuclear magnetic resonance spectra ($^1$H and $^{13}$C) were recorded with the Bruker AVANCE 400 and 600 spectrometers at 20 °C. The chemical shift of the signals is given in parts per million using the (ppm) scale. The following abbreviations indicate the respective multiplicities: s = singlet, d = doublet, t = triplet, q = quartet, quin = quintet, m = multiplet.

The signals of the solvents used were used as standard for calibration:

| Solvent | δ $^1$H-NMR | δ $^{13}$C-NMR |
|---|---|---|
| $CDCl_3$ | 7.26 | 77.16 |
| dmso-$d_6$ | 2.50 | 39.52 |
| $CD_3OD$ | 3.31 | 49.00 |

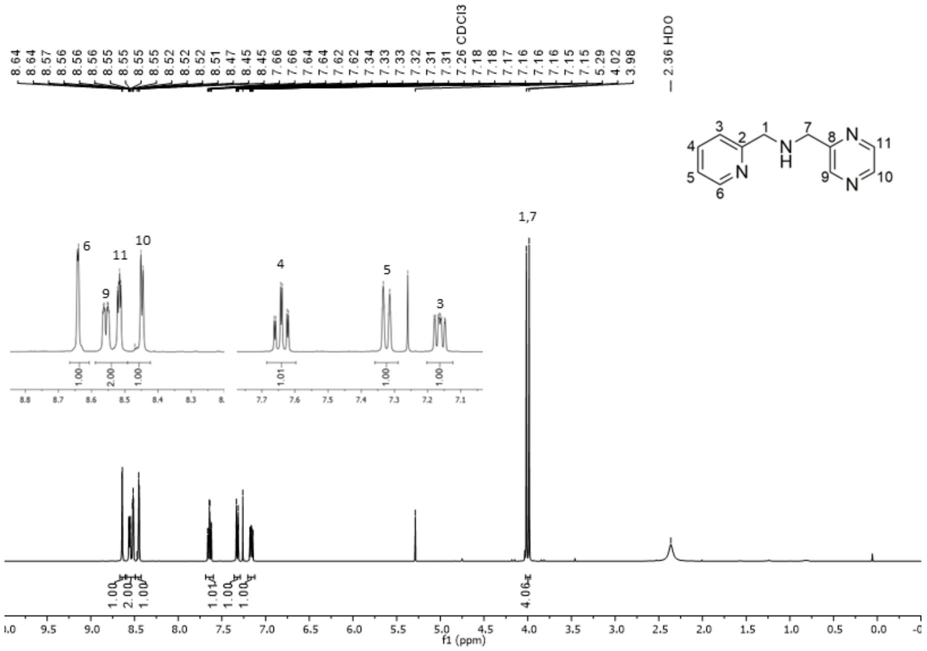


*Figure S1. $^1$H-NMR ($CDCl_3$, 400 MHz) of **1**.*

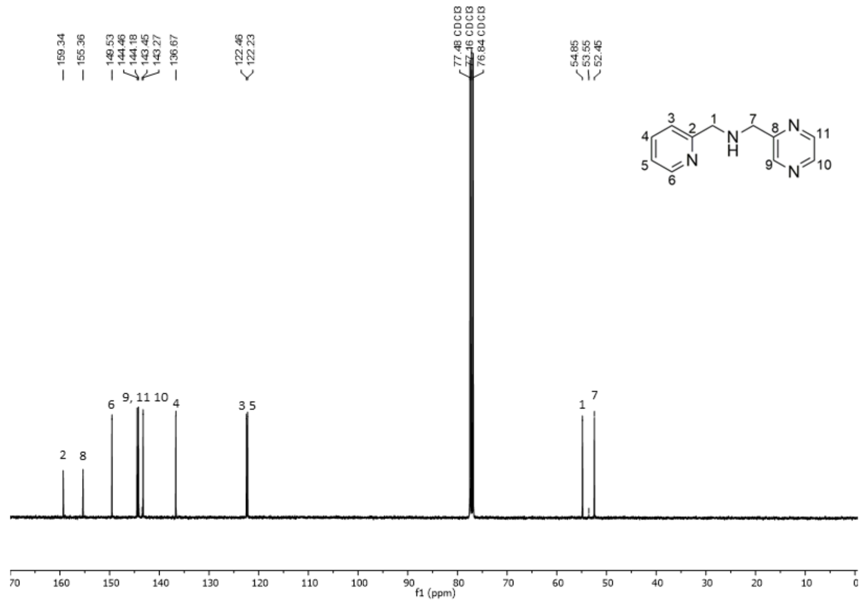


Figure S2. $^{13}$C-NMR ($CDCl_3$, 105 MHz) of **1**.

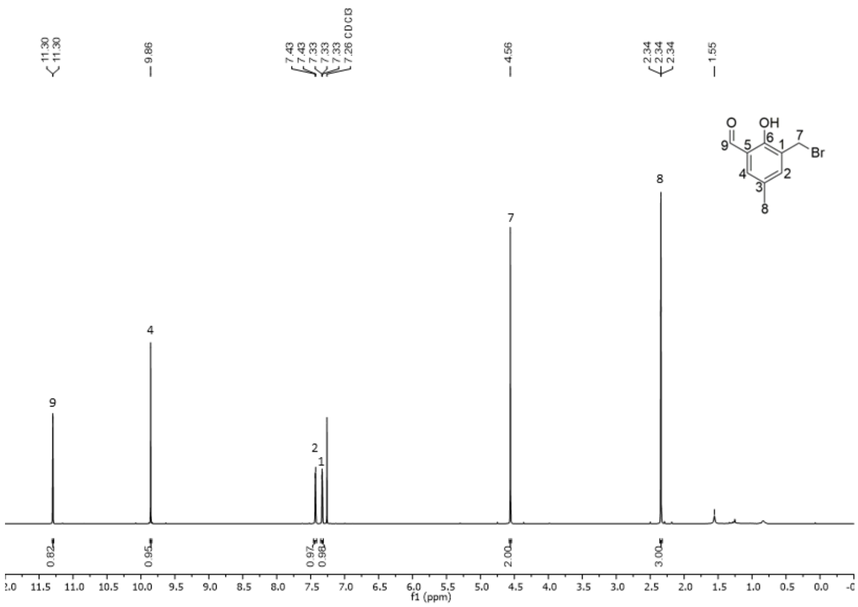


*Figure S3. $^{1}$H-NMR ($CDCl_3$, 400 MHz) of* ***2****.*

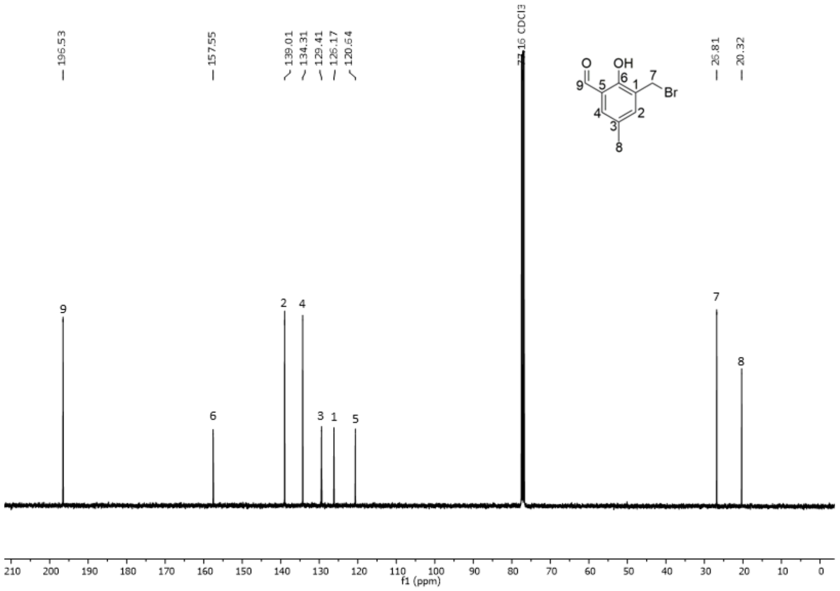

*Figure S4. $^{13}$C-NMR ($CDCl_3$, 105 MHz) of **2**.*

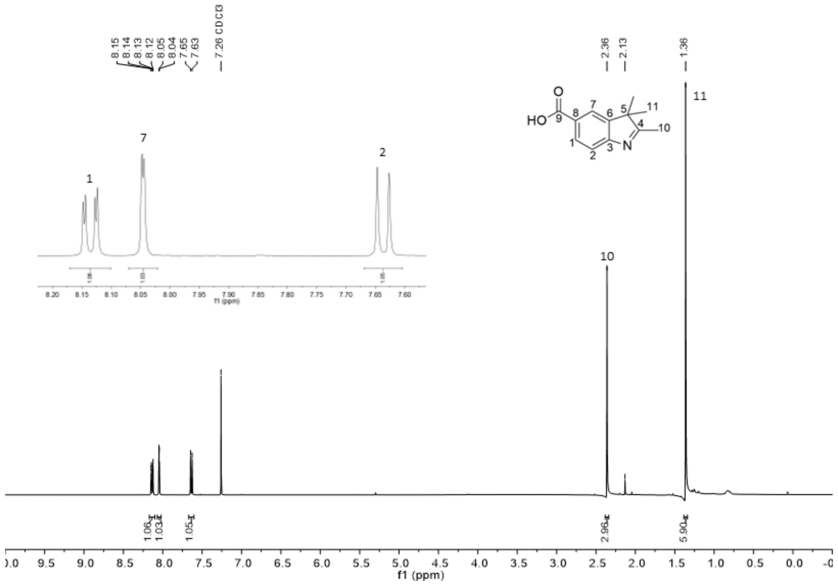

*Figure S5. $^{1}$H-NMR ($CDCl_3$, 400 MHz) of **3**.*

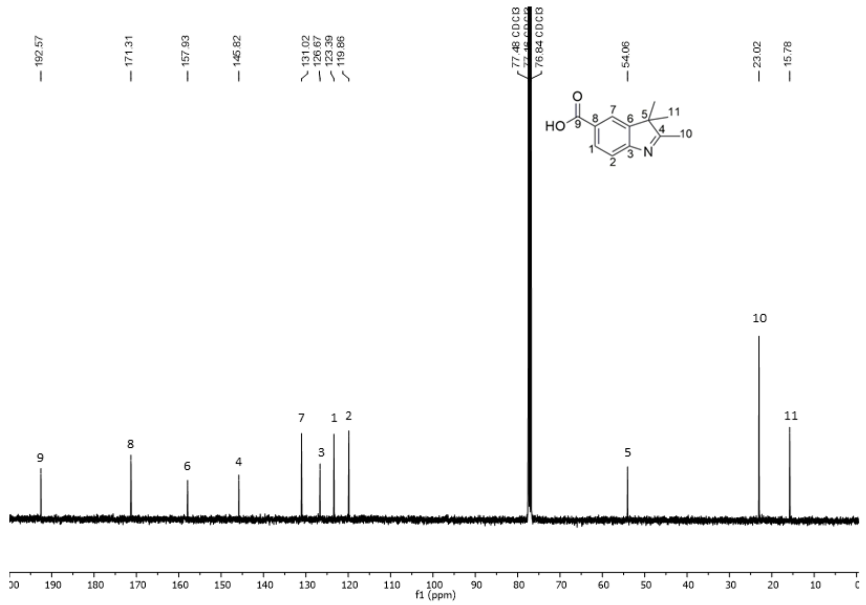

Figure S6. $^{13}$C-NMR ($CDCl_3$, 105 MHz) of **3**.

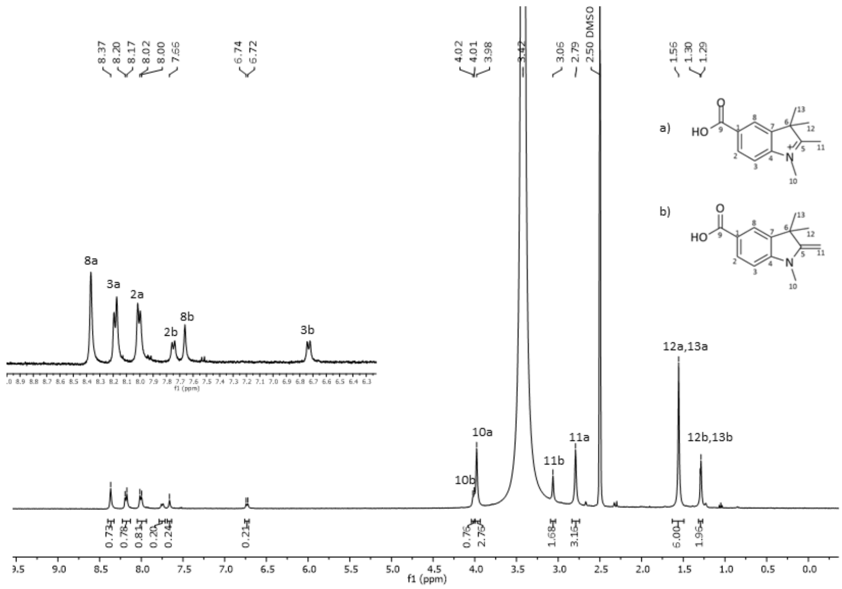

*Figure S7. $^{1}$H-NMR (dmso-$d_6$, 400 MHz) of **4**.*

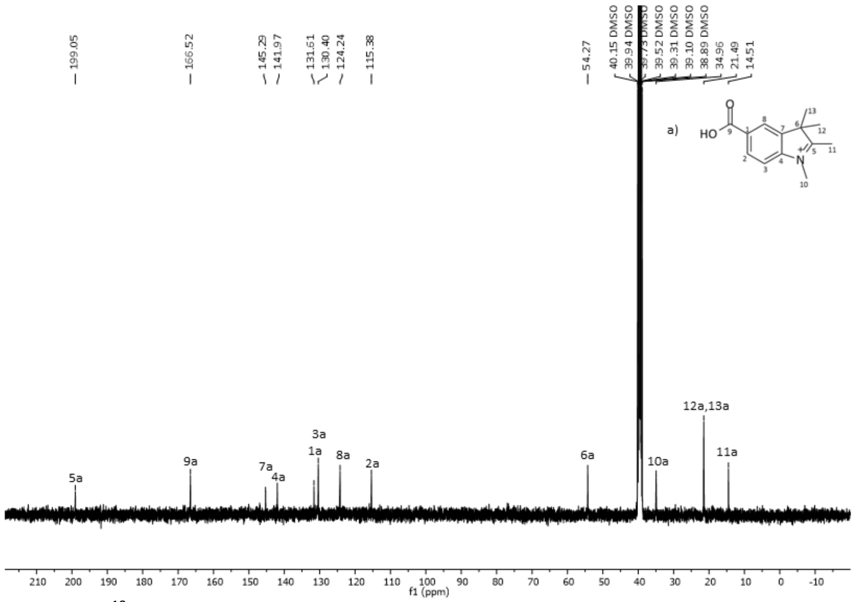

Figure S8. $^{13}$C-NMR (dmso-$d_6$, 105 MHz) of **4**.

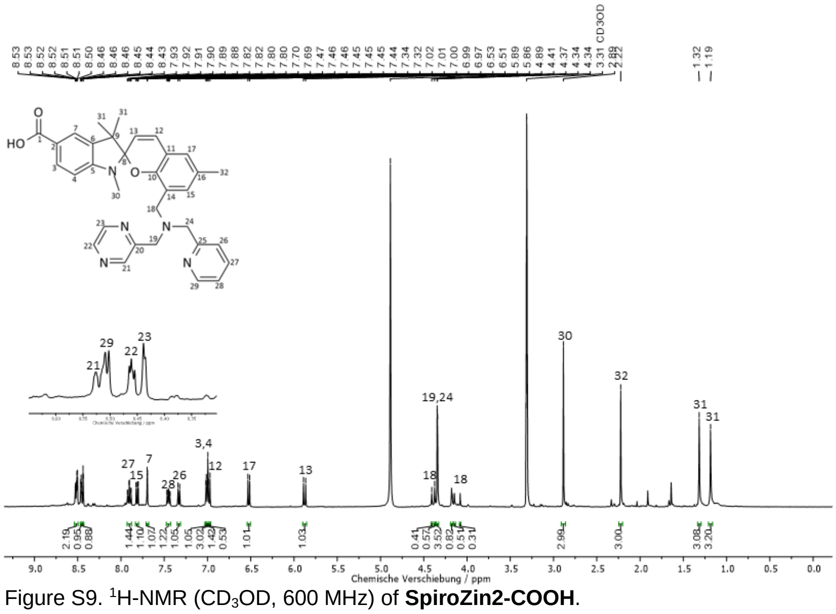

Figure S9. $^{1}$H-NMR ($CD_3OD$, 600 MHz) of **SpiroZin2-COOH**.

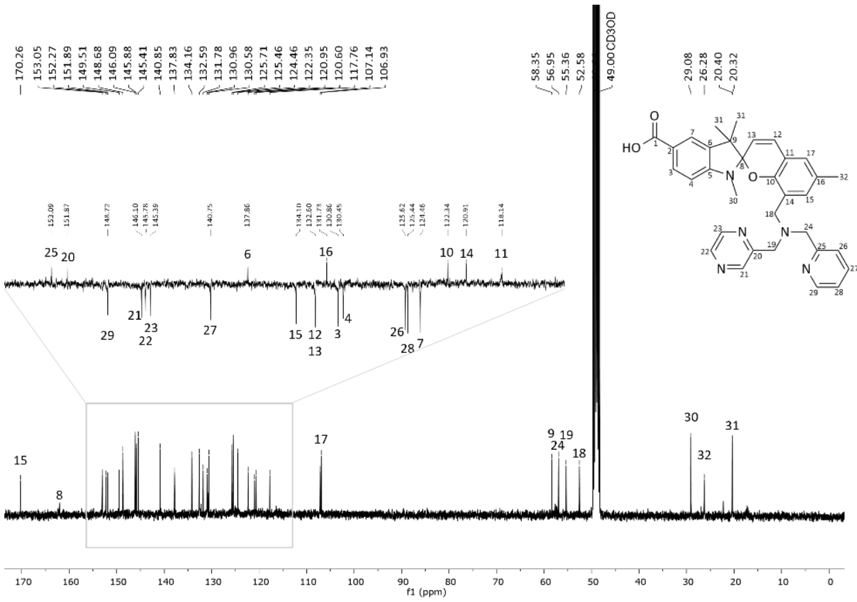


Figure S10. $^{13}$C-NMR ($CD_3OD$, 105 MHz) of **SpiroZin2-COOH**, inset: $^{13}$C-APT-NMR spectrum ($CD_3OD$, 105 MHz).

### 1.3 IR Spectroscopy

The spectra shown were measured with the Fourier transform infrared spectrometers FT/IR-6000 from Jasco FT1000 from Perkin Elmer. The measurements were performed on an ATR crystal. The indicated wavenumbers of the absorption bands are given in $cm^{-1}$.

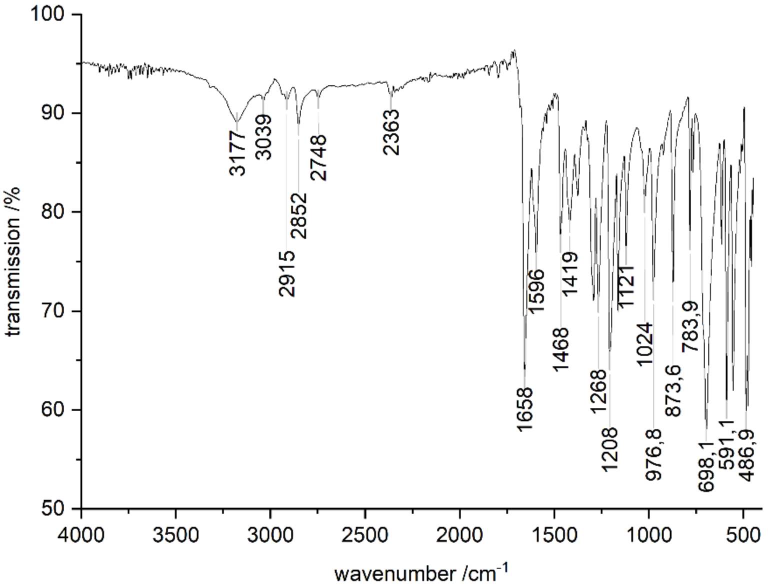


Figure S11. ATR IR spectrum of **1**.

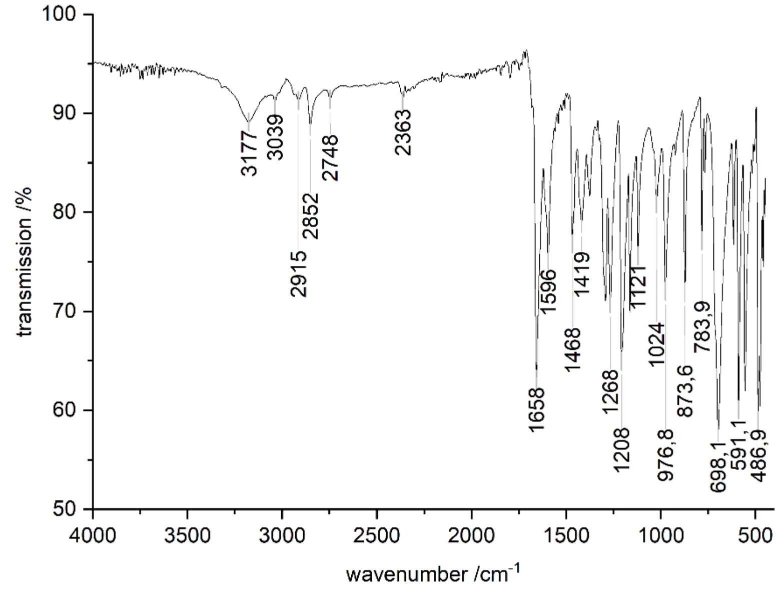


Figure S12. ATR IR spectrum of **2**.

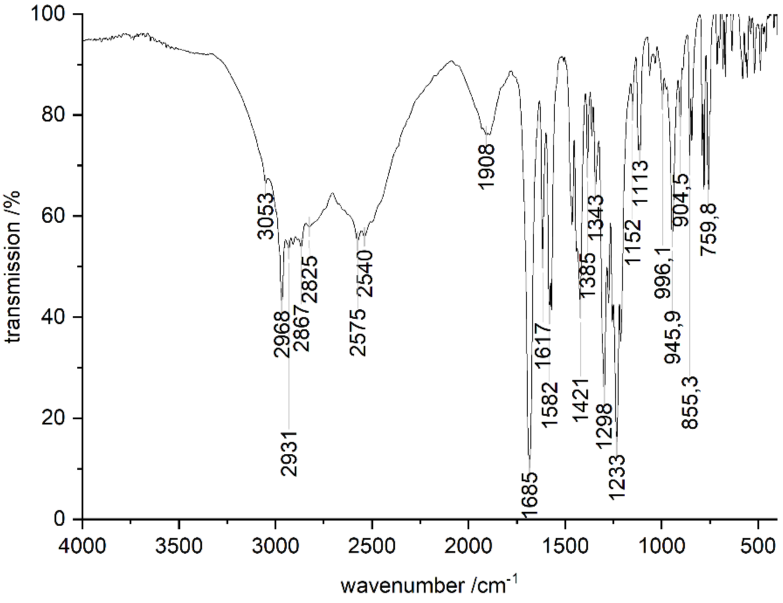


Figure S13. IR spectrum of **3**.

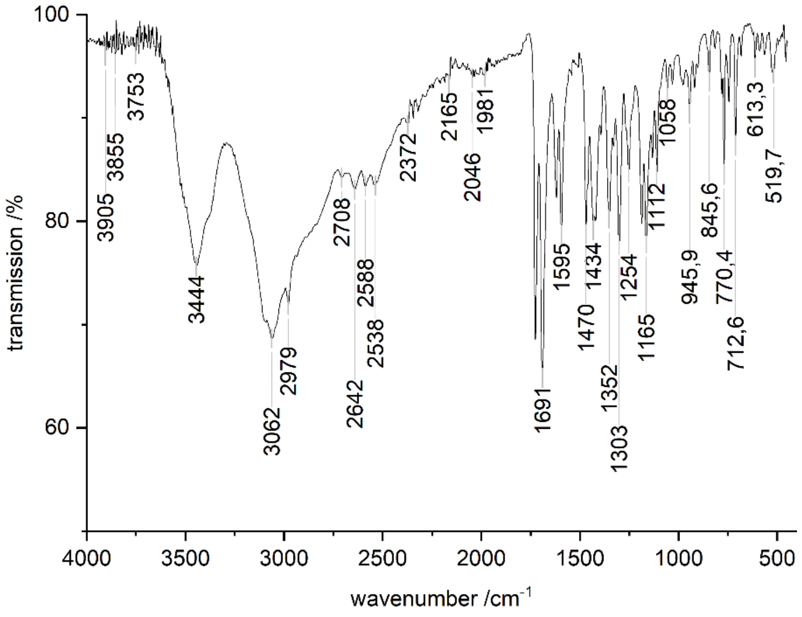


Figure S14. ATR IR spectrum of **4**.

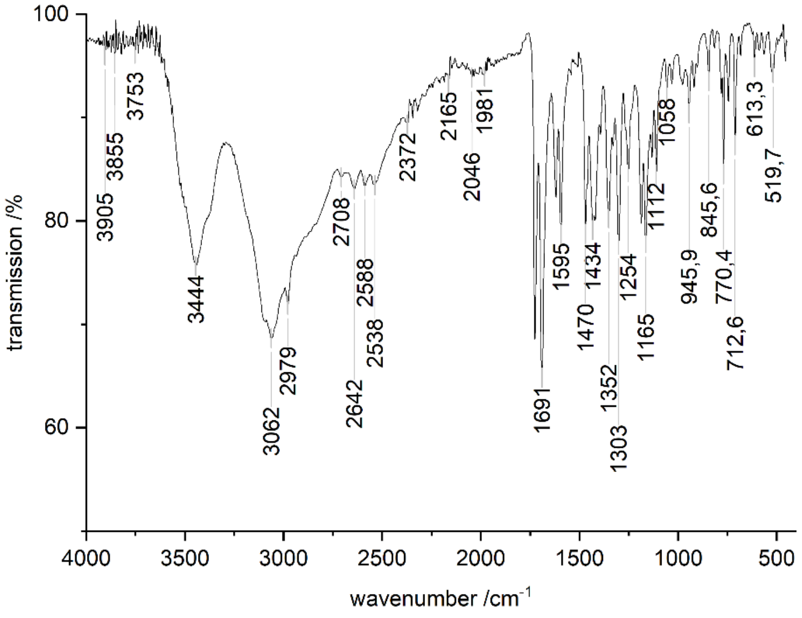


Figure S15. ATR IR spectrum of **SpiroZin2-COOH**.

### 1.4 Mass Spectrometry

The electrospray ionization mass spectrometry measurements were performed by Maximilian Huber, M.Sc. and Dipl. Chem. Michael Lembach (research group of Prof. Dr. Dr. G. Niedner-Schatteburg, RPTU Kaiserslautern-Landau) on a Bruker AmaZon SL spectrometer.

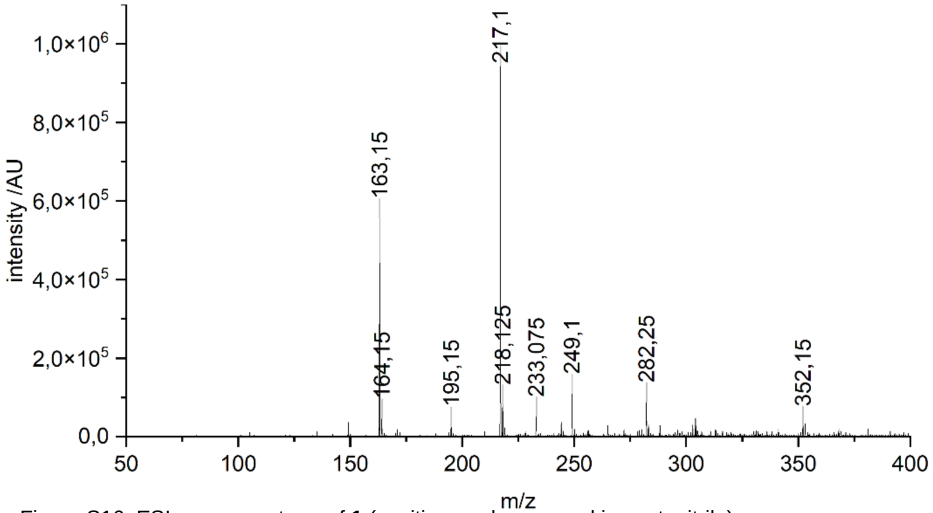


Figure S16. ESI mass spectrum of **1** (positive mode, sprayed in acetonitrile).

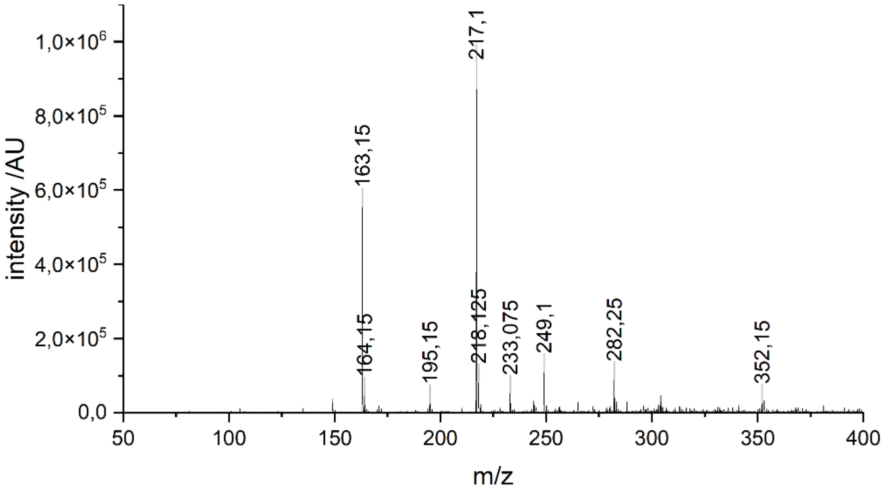


Figure S17. ESI mass spectrum of **2** (positive mode, sprayed in acetonitrile).

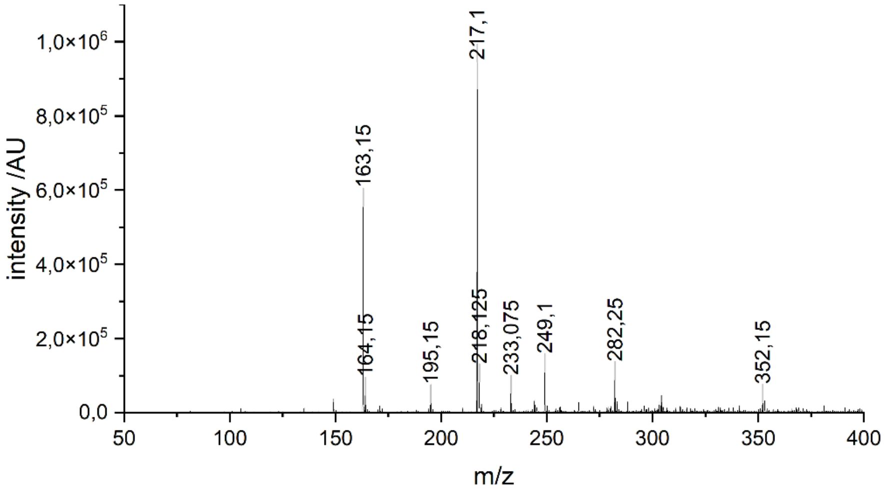


Figure S18. ESI mass spectrum of **3** (positive mode, sprayed in acetonitrile).

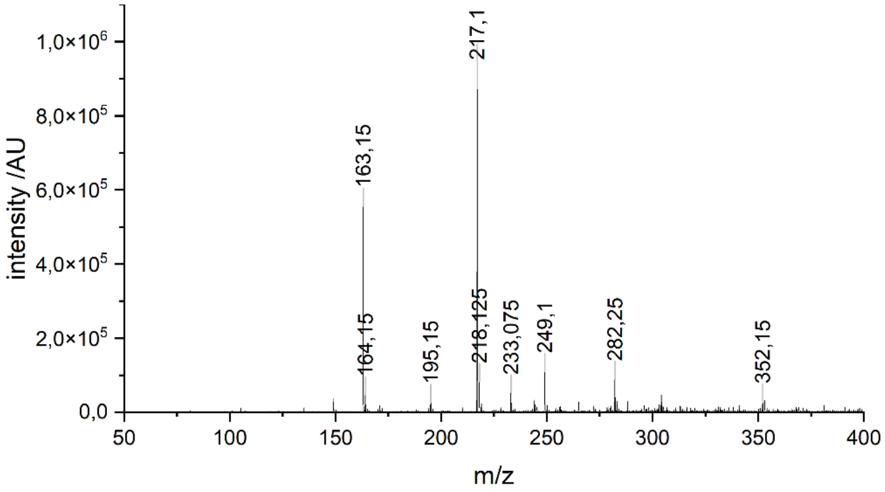


Figure S19. ESI mass spectrum of **4** (positive mode, sprayed in acetonitrile).

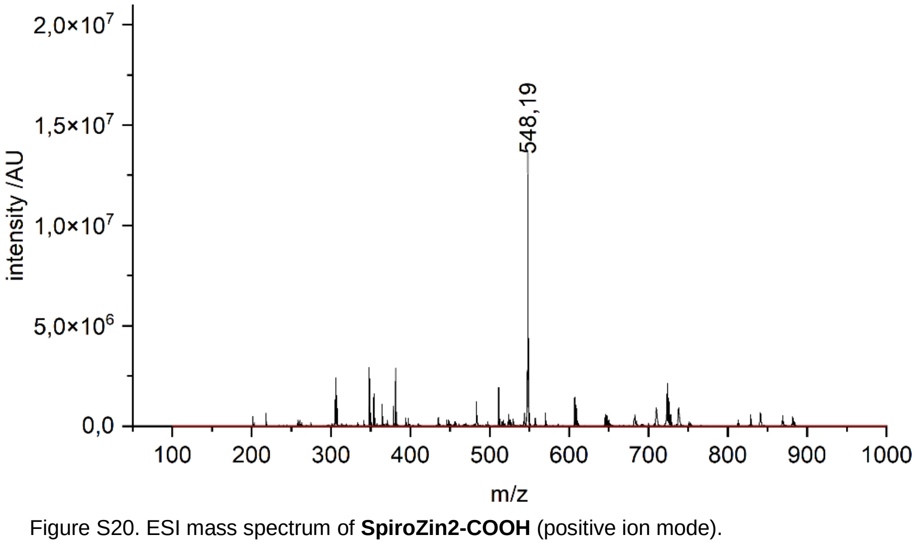


Figure S20. ESI mass spectrum of **SpiroZin2-COOH** (positive ion mode).

## 1.5 Chromatographic purification methods

**MPLC**

The Interchim MPLC system puriFlash PF_XS_20_UV800 was used for purification of crude products. Depending on the amount of crude product, the compound was purified as dryload coated on celite powder. The dryload types PF-DLE-F0004, PF-DLE-F0012, and PF-DLE-F0060 were used. For normal phase purification, the column types PF-50SHIP-F0060, PF-30SIHP-F0040, and PF-30SIHP-F0012 were used. Reversed-phase purification was carried out with the column type PF-15PHC4-F0012.

Table S1. Solvent gradient used for the purification of **1** with column PF-30SIHP-F0040; flowrate 26 mL/min.

| Time /min | Ethyl acetate | ethanol |
|---|---|---|
| 0.00 | 100 | 0 |
| 12:20 | 100 | 0 |
| 20:16 | 90 | 10 |
| 32:26 | 85 | 10 |
| 40:32 | 60 | 40 |
| 50:40 | 60 | 40 |

Table S2. Solvent gradient used for the purification of **3** with column PF-50SIHP-F0120; flowrate 46 mL/min.

| Time /min | pentane | Ethyl acetate |
|---|---|---|
| 0.00 | 96.2 | 3.8 |
| 07:08 | 95.1 | 4.9 |
| 17:49 | 88.5 | 11.5 |
| 32:04 | 63.9 | 36.1 |
| 42:45 | 38.1 | 61.9 |
| 58:47 | 38.1 | 61.9 |
| 1:03:46 | 20 | 80 |

**HPLC**

An Agilent HPLC-1260 Infinity II device was used. Semi-preparative purification of SpiroZin2-COOH was carried out using the column US5PHC4-250/100 from Interchim. Control of the compound's purity was performed using the analytical column US5PHC4-250/46. Related UV/Vis spectra were recorded using the internal diode array detector (series 1200 Infinity, Agilent).

Table S3. Solvent gradient used for the purification of SpiroZin2-COOH with column US5PHC4-250/46.

| Time /min | $H_2O$ + 0.1 % TFA /% | $CH_3CN$ + 0.1 % TFA /% |
|---|---|---|
| 0.10 | 90 | 10 |
| 2.00 | 90 | 10 |
| 8.00 | 50 | 50 |
| 20.00 | 0 | 100 |
| 21.00 | 0 | 100 |
| 22.00 | 90 | 10 |
| 25.00 | 90 | 10 |

Table S4. Solvent gradient used for the purification of SpiroZin2-COOH with column US5PHC4-250/100.

| Time /min | $H_2O$ + 0.1 % TFA /% | $CH_3CN$ + 0.1 % TFA /% |
|---|---|---|
| 0.10 | 90 | 10 |
| 5.00 | 90 | 10 |
| 30.00 | 0 | 100 |
| 33.00 | 0 | 100 |
| 36.00 | 90 | 10 |
| 40.00 | 90 | 10 |

Table S5. Solvent gradient used for the purity control of SpiroZin2-COOH with column US5PHC4-250/46.

| Time /min | $H_2O$ + 0.1 % TFA /% | $CH_3CN$ + 0.1 % TFA /% |
|---|---|---|
| 0.10 | 90 | 10 |
| 5.00 | 90 | 10 |
| 30.00 | 0 | 100 |
| 33.00 | 0 | 100 |
| 36.00 | 90 | 10 |
| 40.00 | 90 | 10 |

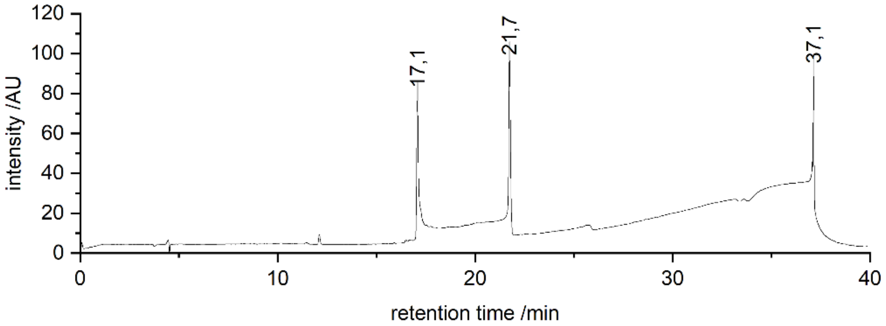


Figure S21. Chromatogram of SpiroZin2-COOH (wavelength 250 nm). Two signals are obtained for SpiroZin2-COOH caused by the equilibrium of the zwitterionic MC form (RT = 17.1 min) and the closed SP form (RT = 21.7 min). Method used as described in Table S5. Peak at RT = 37.1 min is caused by the column.

# 2. Photophysical Properties of SpiroZin2-COOH

### 2.1 UV/vis Spectroscopy

The UV/Vis spectra were measured with an Agilent Technologies spectrometer Cary 60 at 25°C. Quartz glass cuvettes with a pathlength of 10 mm were used.

### 2.2 Absorption Spectra

SpiroZin2-COOH (5 µM, stock solution: 2.18 mM in DMSO) was measured in PIPES buffer (2 mL) (Figure S23). $Zn^{2+}$ (100 eq., 500 mM) was added as $ZnSO_4 \cdot 7H_2O$ solution (c = 0.5 M in $H_2O_{dd}$). The color visibly changed from yellow to a bright pink (Figure S23).

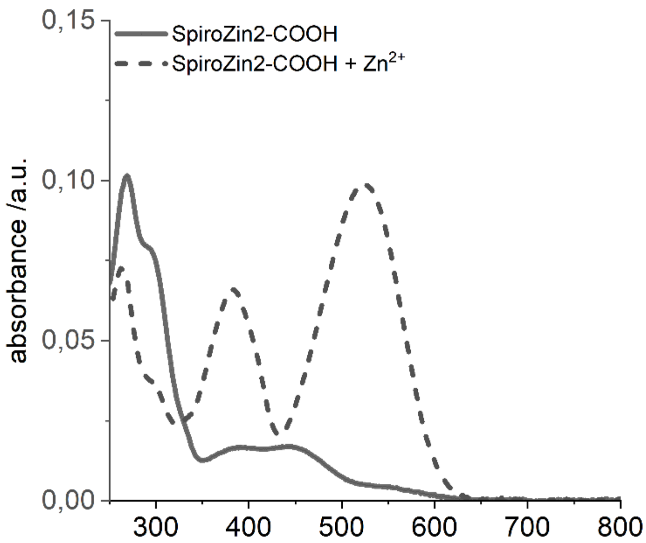


Figure S22. Absorption spectra of SpiroZin2-COOH in PIPES-buffer before (solid line) and after addition of $ZnSO_4 \cdot 7H2O$ (100 eq., dashed line).

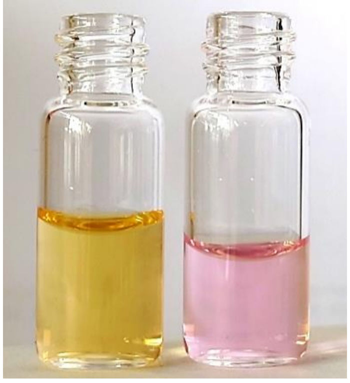

Figure S23. SpiroZin2-COOH solution in PIPES-buffer before (left; yellow) and after addition of a $ZnSO_4{\cdot}7H_2O$ solution (right; bright pink).

### 2.3 Extinction Coefficient

The extinction coefficient was determined by a combined measurement of $^1$H-NMR (DMSO-$d_6$, 400 MHz, D1 = 30) and UV/vis spectroscopy. First, the concentration of a sample was determined via NMR spectroscopy. Then, this sample was used to prepare stock solutions with different concentrations, which were investigated via UV/Vis spectroscopy.

**Determination of the sample's concentration**. A small amount of SpiroZin2-COOH was dissolved in 450 µL DMSO-$d_6$ and 1µL of cyclohexane was added as a reference substance. One part of this solution was used for the $^1$H-QNMR-measurement (Figure S24). A comparison of the integration of a SpiroZin2-COOH proton (Figure S24, left box) to the integration of the cyclohexane (right box), whose concentration is known, yields the concentration of SpiroZin2-COOH.

**Determination of the extinction coefficient of Zn-SpiroZin2-COOH**. For this, different amounts of the stock solution of the previous, through H-QNMR determined concentration were dissolved in PIPES. After addition of an excess of $Zn^{2+}$ ($ZnSO_4{\cdot}7H_2O$, 0,5 M in $H_2O_{dd}$), the absorbance at 524 nm was determined for each sample (Figure S25). Using Lambert-Beer's law, the extinction coefficient for each titration step was calculated and later the values were averaged.

$$\mathbf{E_\lambda = \varepsilon_\lambda \cdot c \cdot d;\ with\ d = 1\ cm}$$

This procedure was carried out several times. It was found that the values of the extinction coefficient differ slightly depending on which peak in the NMR is used as a reference signal to cyclohexane. In addition, the integration of cyclohexane cannot be set precisely due to the broadening of the peak. An average value of the coefficients obtained was therefore used for further concentration determinations of stock solutions. ($\varepsilon_{524} = 2{,}6(8) \cdot 10^4$ $Lcm^{-1}mol^{-1}$).

All values obtained: $\varepsilon_{524} = 1.7 \cdot 10^4$ $Lcm^{-1}mol^{-1}$; $\varepsilon_{524} = 2.7 \cdot 10^4$ $Lcm^{-1}mol^{-1}$; $\varepsilon_{524} = 3.4 \cdot 10^4$ $Lcm^{-1}mol^{-1}$.

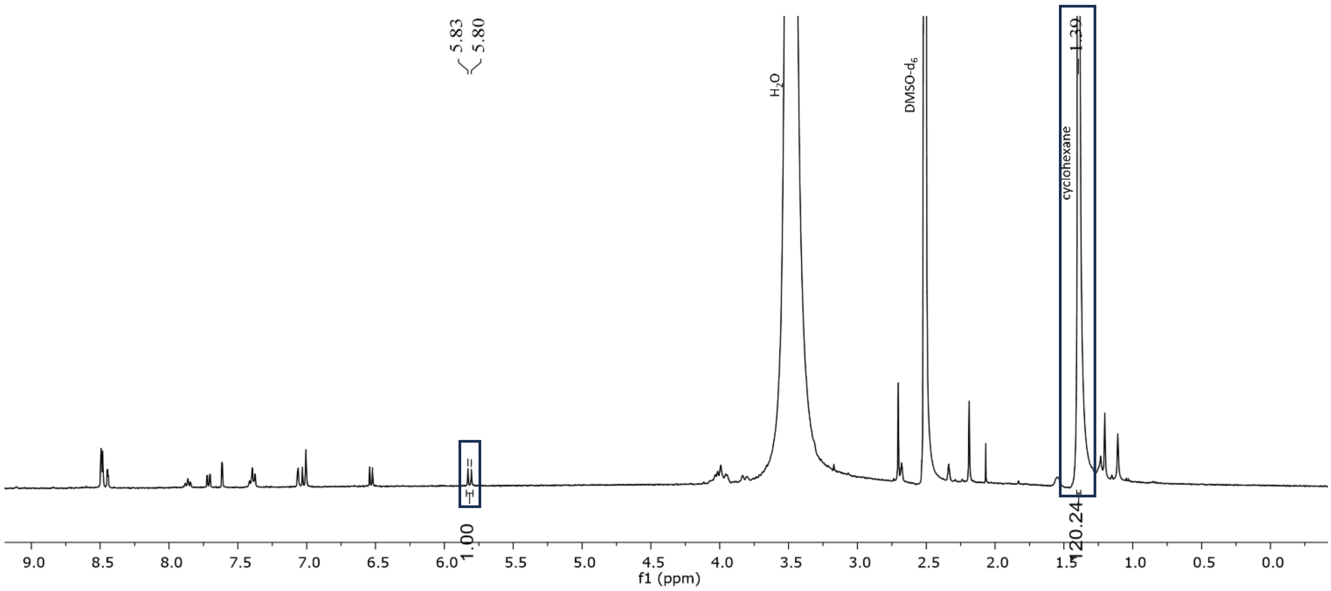


Figure S24. $^1$H-QNMR spectrum of SpiroZin2-COOH and cyclohexane as a reference substance (DMSO-$d_6$, 400 MHz, D1 = 30).

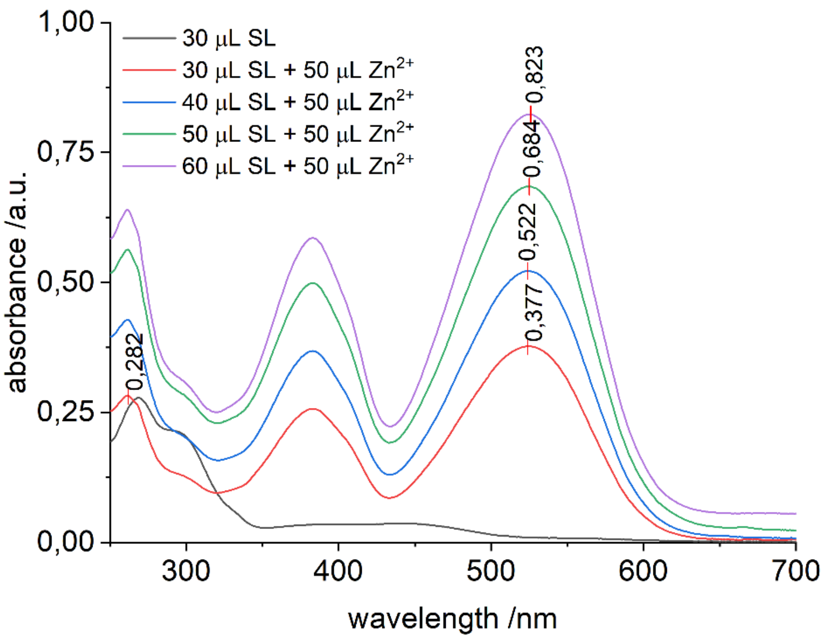


Figure S25. UV/vis spectra of different concentrations of SpiroZin2-COOH stock solution (SL) in PIPES buffer at 25 C.

### 2.4 Fluorescence Spectroscopy

The fluorescence spectra were measured with a fluorescence spectrometer FL 8500 from Perkin Elmer. The cuvettes used were either made of plastic or quartz glass, had a pathlength of 10 mm, and a transmittance of 200 nm to 2500 nm. The excitation wavelength was 518 nm, whereas emission was recorded in the range 550nm-900nm. The slit width was defined as 20 nm and a voltage of 550 V was used.

### 2.5 Emission Spectra

The measurements were carried out at 25 °C while the solution was continuously stirred. The sample was prepared in DMSO and the concentration was determined via absorption spectroscopy ($\varepsilon_{524}$ = 2.6(8) · $10^4$ $Lmol^{-1}cm^{-1}$, c = 5 µM). A buffer solution (PIPES, pH 7, 2.0 mL) was used as solvent. To investigate the turn-on, 500 µM of a $ZnSO_4 \cdot 7H_2O$ solution (c = 0.5 M, in $H_2O_{dd}$) were added. The emission in the range from 650 to 725 nm before and after addition of $Zn^{2+}$ was integrated and the values were offset against each other to obtain the turn-on (here turn-on: 20; up to 38).

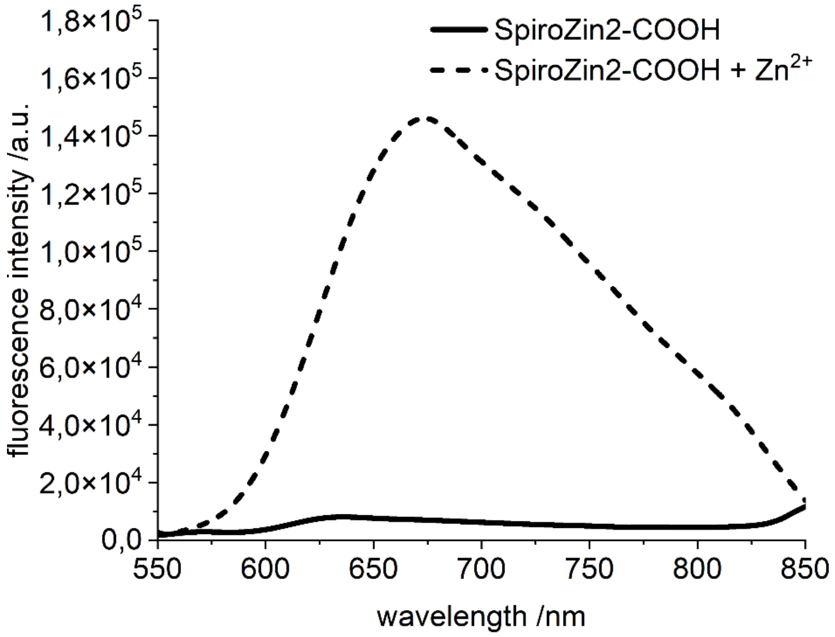


Figure S26. Emission spectra of SpiroZin2-COOH before and after addition of $ZnSO_4 \cdot 7H_2O$ (100 eq.) in PIPES buffer at 25 C, $\lambda_{ex}$ = 518 nm.

### 2.6 Affinity Studies

To determine the dissociation constant ($K_d$) of SpiroZin2-COOH, titration studies were performed. PIPES (1.5 mL) was prepared with 1 mM $Na_4EGTA$ (ethylene glycol tetraacetic acid tetra sodium salt) and 5 µM SpiroZin2-COOH. $ZnSO_4\cdot 7H_2O$ (0.5 M in water) was added in 2 µL steps to achieve nanomolar concentration ranges of buffered free zinc. The actual amount of buffered free zinc was later determined using the web based program MaxChelator (https://somapp.ucdmc.ucdavis.edu/pharmacology/bers/maxchelator/webmaxc/webmaxcS.htm). Emission spectra were then plotted and integration values (650 – 725 nm) were normalized to the titration maximum (Figure S27, left). Normalized fluorescence intensity (y) were plotted against buffered free zinc concentrations (x). A non-linear fit to the following equation was performed to determine $K_d$ to 3.1 nM with $R^2$ = 0.984 (Figure S27, right). The measurement was repeated two more times. Here, the $K_d$ was determined to 4,1 nM with $R^2$ = 0,983 and to 2,1 nM with $R^2$ = 0,895. The average value is therefore $K_d$ = 3.1 nM.

$$\mathbf{y = (B\cdot x)/(K_d+x) \text{ with } B = 1}$$

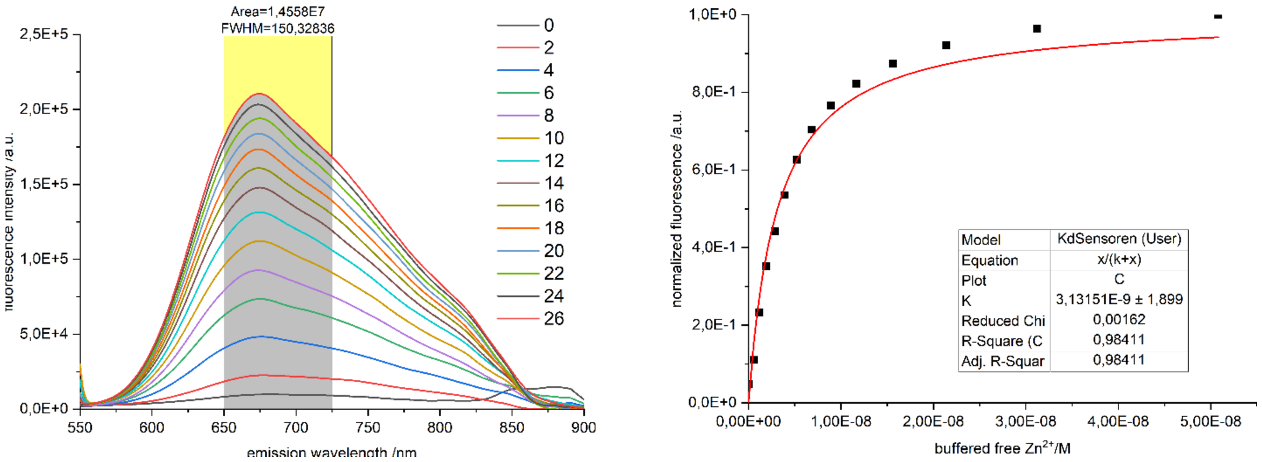


Figure S27. Left: Emission spectra of zinc titration to SpiroZin2-COOH (5 µM) and $Na_4EGTA$ (1 mM) in PIPES buffer at 25 °C, $\lambda_{ex}$ = 518 nm. Right: Normalized fluorescence intensity of these spectra plotted against concentration of buffered free zinc.

### 2.7 Selectivity Studies

Measurements were carried out at 25 °C in PIPES buffer. 5 µL of a 1.15 mM aqueous SpiroZin2-COOH solution was added to the buffer system ($c_{final}$ = 2.30 µM). First, 100 eq. of one of the metal salts listed below (each 1.15 µL, for $Na_2HPO_4$ only 0.57 µL; 0.5 M, in water) was added and the emission spectrum of the resulting solution was recorded. Then, 100 eq. $Zn^{2+}$ (1.15 µL $ZnSO_4\cdot 7H_2O$ in water, 0.5 M) was added and a second emission spectrum was recorded.

Measurements with $Fe_2Cl$ and $[(CH_3CN)_4Cu]PF_6$ were carried out in degassed PIPES. The stock solutions were prepared in dry and degassed acetonitrile under inert conditions.

Tested metal salts: $Na_2HPO_4\cdot H_2O$ (>99%); $MgCl_2\cdot 6H_2O$ (>99%); $CaCl_2$ (>94%); $CdCl_2$ (>99%); $NiCl_2\cdot 6H_2O$ (>98%); $CuCl_2\cdot 6H_2O$ (>98%); $MnCl_2\cdot 4H_2O$ (>98%); $FeCl_3\cdot 6H_2O$ (>98%); $FeCl_2$ (>99%), $[(CH_3CN)_4Cu]PF_6$ (>98%).

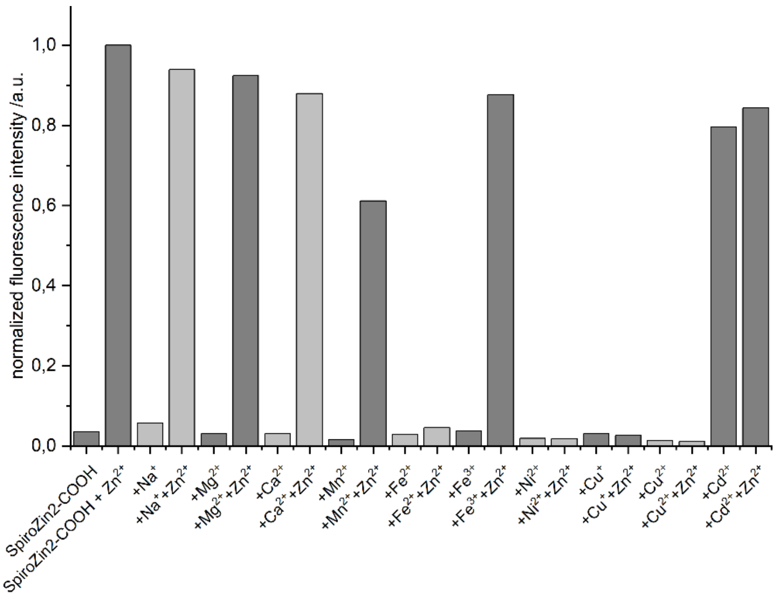


Figure S28. Comparison of the turn-on of various metal salt solutions with SpiroZin2-COOH. (PIPES buffer, pH 7). Turn-on after addition of $Zn^{2+}$: $Na^{+}$: 16.5; $Mg^{2+}$: 30.5; $Ca^{2+}$: 28.7; $Mn^{2+}$: 38.3; $Fe^{2+}$: 1.54; $Fe^{3+}$: 23.4; $Ni^{2+}$: 0.96; $Cu^{+}$: 0.88; $Cu^{2+}$: 0.85; [$Cd^{2+}$]: 1.1.

### 2.8 pH Dependency

Buffer solutions of pH 3 – 6 were prepared using citric acid and sodium phosphate (Table 6). PIPES was used for pH 7. Buffer solutions of pH 8−10 were prepared using ammonium and ammonia (Tabe 7) in water. At 25 °C and continuous stirring, a SpiroZin2-COOH solution (c = 1.15 mM) was added and measured. Afterwards, 100 eq. of $Zn^{2+}$ ($ZnSO_4 \cdot 7H_2O$, c = 0.5 M) were added and the measurement was repeated.

Table S6. Preparation of buffer solutions of citric acid and sodium phosphate. Stock solution of citric acid and sodium phosphate (0.1 M) were prepared. The acid and base were mixed in the shown rations to receive pH values 3 to 6. Afterwards, pH values were checked and corrected by adding the basic stock solution.

| pH | p*K*s used for calculations | Volume acidic stock solution /mL | Volume basic stock solution /mL |
|---|---|---|---|
| **3** | 3.14 | 8.72 | 6.28 |
| **4** | (3.14+4.77)/2 | 7.07 | 7.93 |
| **5** | 4.77 | 5.50 | 9.50 |
| **6** | (4.77+6.39)/2 | 4.13 | 10.87 |

Table S7. Preparation of buffer solutions of ammonia and ammonium chloride in 100 mL water. The acid and base were mixed in the shown rations to receive pH values 8 to 10. Afterwards, pH values were checked and corrected by adding HCl or $NH_3$ in water.

| pH | Volume of $NH_3$ (25% aq.) /µL | $NH_4Cl$ /mg |
|---|---|---|
| **8** | 39 | 500 |
| **9** | 270 | 345 |
| **10** | 621 | 91 |

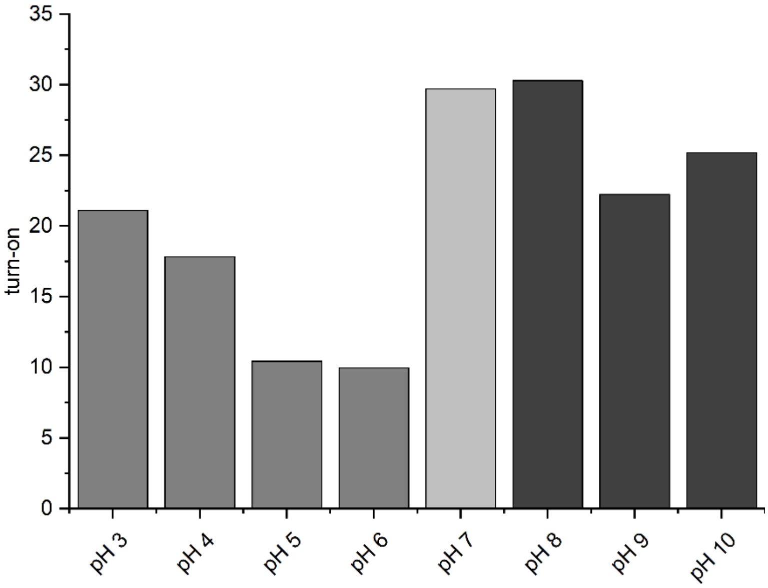


Figure S29. Turn-on of SpiroZin2-COOH after addition of zinc at different pH values. Measurements at pH 3-6 were carried out in aqueous citric acid/sodium phosphate buffer solutions; that of pH 7 was carried out in PIPES, and those at pH 8-10 in aqueous ammonium/ammonia buffer solution. Turn-on: pH 3: 21.1; pH 4: 17.8; pH 5: 10.4; pH 6: 9.9; pH 7: 29.7; pH 8: 30.3; pH 9: 22.2; pH 10: 25.2.

### 2.9 Quantum Yield

As the photophysical properties of tetraphenylporphyrine (TPP, Φ = 0.11) match the expected properties of SpiroZin2-COOH, TPP was used as reference to determine the quantum yield of SpiroZin2-COOH.[4]

Measurements were carried out in acetonitrile at 25 °C. 550 nm was used as excitation wavelength for the fluorescence measurements. Further parameters were defined as follows; slit width 20 nm and voltage 550 V.

Both substances, TPP and SpiroZin2-COOH (metal-free and zinc-bound state), were measured at different concentrations. To remove contaminating zinc from the acetonitrile solvent, EDTA (70µM, ~2 eq. of final probe concentration) was added to the solution used for metal-free SpiroZin2-COOH.

Absorption spectra and emission spectra were measured successively during a titration series (Figure S30). For each substance, the measurements were performed two independent times.

Absorption values at 550 nm and fluorescence integration values (600 – 800 nm) were determined for each measurement. The absorption maxima at 550 nm were then plotted against the integration of the fluorescence intensity and linear fits were calculated (Figure S31). The slope of these fits were used in the following equation to calculate the quantum yield.

As SpiroZin2-COOH is non-fluorescent, the values obtained fall into the background noise, so that a linear fit as described above is not meaningful (Figure S31, top left). Accordingly, the quantum yield of the zinc-free state cannot be determined reliably.

For zinc-bound SpiroZin2-COOH, because of the independence of the individual measurements of both substances, the resulting slopes could be mathematically related to each other. This way, **four** values were obtained, which were then averaged.

$$\Phi_{sample} = \Phi_{standard} \times (slope_{sample} / slope_{standard}) \times (\eta_{sample}/\eta_{standard})$$

The refractive indices used for calculations were $\eta_{sample;\ acetonitrile}$ = 1.344 and $\eta_{standard,\ toluene}$ = 1.4969. Measurements were repeated two times and the values were averaged to obtain the quantum yield of $\Phi_{Zn\text{-}SpiroZin2\text{-}COOH}$ = 0.0065 (6).

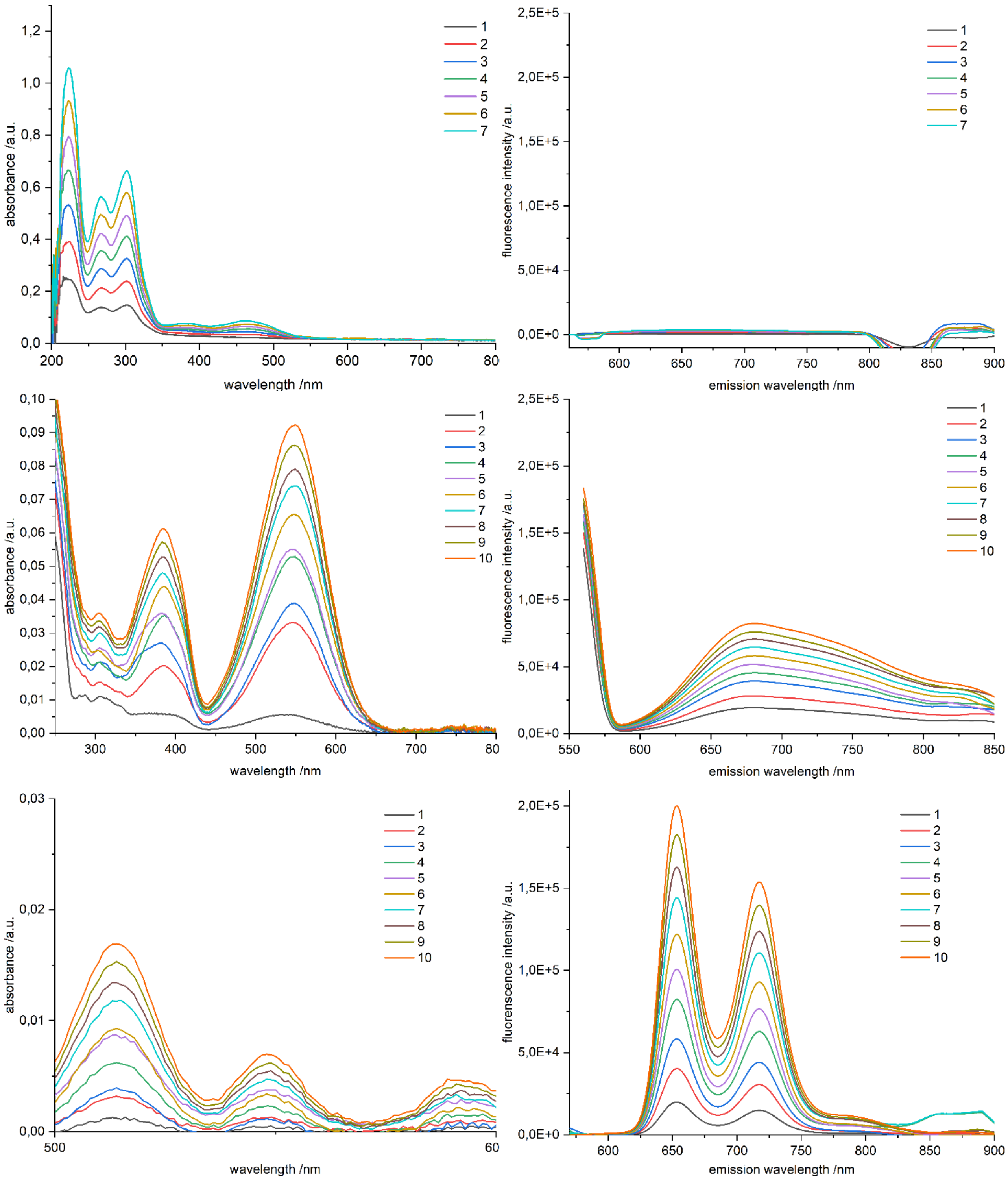


Figure S30. Left column: absorption measurements of SpiroZin2-COOH (top), SpiroZin2-COOH + $Zn^{2+}$ (middle), and TPP (bottom). Right column: corresponding emission measurements of SpiroZin2-COOH (top); SpiroZin2-COOH + $Zn^{2+}$; (middle), and TPP (bottom).

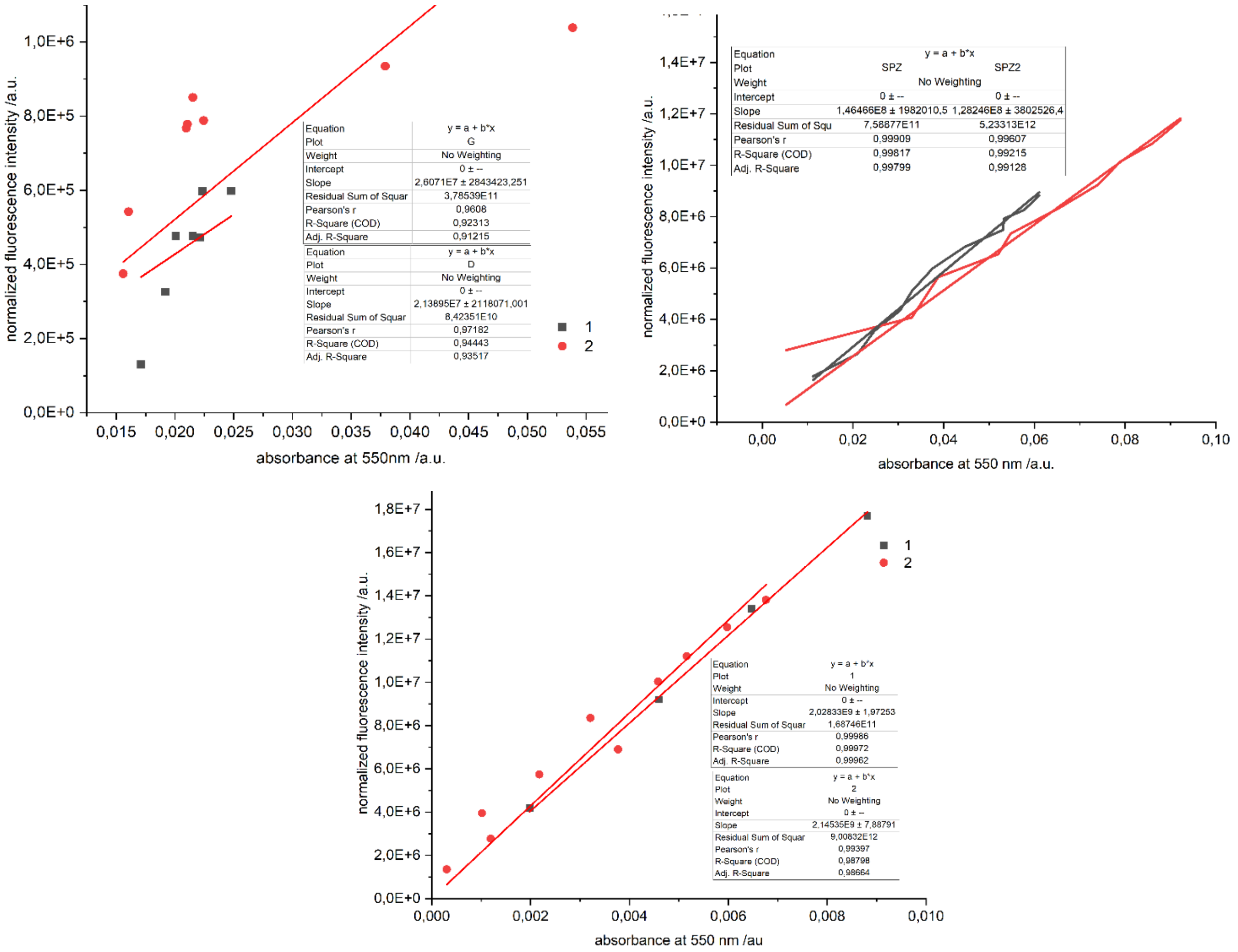


Figure S31. Absorption values at 550 nm plotted against normalized fluorescence intensities (integration 600 – 800 nm) of two individual measurements for each substance. Linear fits in red. Top left: SpiroZin2-COOH (metal-free state), top right: SpiroZin2-COOH + $Zn^{2+}$; bottom: TPP.

## 3. Cell Culture and Live Cell Imaging

**General Materials and Methods**

HeLa cells were cultivated in 75 $cm^2$ cell culture flasks at 37 °C, 5 % $CO_2$ and 97 % relative humidity. The cells were cultured in DMEM (1X) (Dulbecco's modified Eagle medium; Gibco Life Technologies, Germany) supplemented with 10% FBS (fetal bovine serum; PAN-Biotech, Germany) and antibiotics (100 U/mL penicillin and 100 µg/mL streptomycin). For incubation procedures, DMEM was used without the additives. Cell imaging was performed using a confocal laser scanning microscope (LSM900, Zeiss, Oberkochen, Germany) as reported previously.[5]

All studies were repeated three times. For all cell plates, images were taken at different points on the plates in order to rule out uneven or only local distribution of the dyes and suspension rates.

**Live Cell Imaging:**

HeLa cells ($8 \cdot 10^4$ cells/ chamber) were plated onto 8-Well µ-Slides (ibidi, Gräfelfing, Germany) and grown for 20 h to a confluence of about 70 %. The cells were treated with SpiroZin2-COOH (5 µM), zinc pyrithione (50 µM), TPEN (100 µM) or a combination of these substances for 10 min each, as shown in Figure S32. The amount of washing steps and media changes has been equally for all chambers.

Nuclear staining was performed with Hoechst 33342 (20 µM) for 20 min in all four wells. Afterwards, the medium was removed and the cells were carefully rinsed with PBS buffer before SpiroZin2-COOH (10 µM) was added in DMEM. SpiroZin2-COOH was incubated for 10 min. Then the medium was removed and the cells were again rinsed with PBS buffer. Afterwards, zinc pyrithione (100 µM) was added and the cells were incubated for another 10 min. Next, the medium was removed, the cells rinsed with PBS buffer, and TPEN (200µM) was added followed by another incubation time of 10 min. After finishing these preparations, the cells were carefully rinsed

with PBS buffer and DMEM Fluorobrite (Gibco Life Technologies, Germany) was applied to the cells to achieve optimal conditions for confocal fluorescence microscopy.
For the colocalization studies, the same preparation sequence was carried out. Following concentrations were prepared and incubated: 20 µM Hoechst 33342, 5 µM SpiroZin2-COOH and 50 µM zinc pyrithione (Figure S32). LysoTracker Green DND-26 #8783 (Cell Signaling Technology, Germany; 1:10.000, 33nM) was added to the dye-free DMEM Fluorobrite and imaging was carried out immediately. The cells were analyzed using a confocal laser scanning microscope LSM900 (Zeiss, Oberkochen, Germany) equipped with a 63x oil objective (Plan-Apochromat 63x/1.40 DIC M27) as reported previously.[5] The following lasers were used: 405 nm laser (Hoechst 33342), 488 nm laser (LysoTracker Green) and 561 nm laser (SpiroZin2-COOH). Images were acquired using the multi-track mode and processed by ZEN software version 3.4 (Zeiss). All imaging studies were repeated three times.
The images were evaluated using the free software ImageJ (1.53e). For this purpose, the background was subtracted and the fluorescence maxima were determined with a prominence>40. To determine the Pearson's correlation coefficient the ImageJ plugin JACoP v2.0 was used.[6]

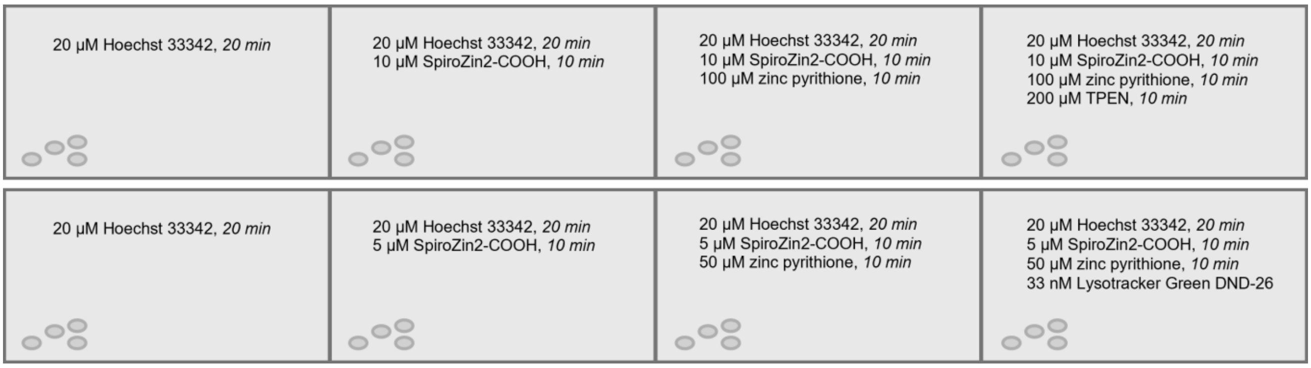

Figure S32. Preparation sequence for live cell imaging. Top: Turn-on studies. Bottom: Colocalization studies.

## References


[1] Rivera-Fuentes, P.; Wrobel, A. T.; Zastrow, M. L.; Khan, M.; Georgiou, J.; Luyben, T. T.; Roder, J. C.; Okamoto, K.; Lippard, S. J. A far-red emitting probe for unambiguous detection of mobile zinc in acidic vesicles and deep tissue. *Chem. Sci*. **2015**, *6*, 1944–1948

[2] Heng, S.; Reineck, P.; Vidanapathirana, A. K.; Pullen, B. J.; Drumm, D. W.; Ritter, L. J.; Schwarz, N.; Bonder, C. S.; Psaltis, P. J.; Thompson, J. G.; Gibson, B. C.; Nicholls, S. J.; Abell, A. D. Rationally designed probe for reversible sensing of zinc and application in cells. *ACS Omega* **2017**, *2*, 6201−6210

[3] Tomasulo, M.; Kaanumal, S. L.; Sortino, S.; Raymo, F.M. Synthesis and properties of benzophenone-spiropyran and naphthalene-spiropyran conjugates. *J. Org. Chem.* **2007**, *72*, 595-605.

[4] Seybold, P. G.; Gouterman, M. Porphyrins: XIII: Fluorescence spectra and quantum yields. *J. Mol. Spectrosc.*, **1969**, *31*, 1−13.

[5] Carlsson, M. J.; Vollmer, A.S.; Demuth, P.; Heylmann, D.; Reich, D.; Quarz, C.; Rasenberger, B.; Nikolova, T.; Hofmann, T. G.; Christmann, M.; Fuhlbrueck, J. A.; Stegmüller, S.; Richling, E.; Cartus, A. T.; Fahrer, J. p53 triggers mitochondrial apoptosis following DNA damage-dependent replication stress by the hepatotoxin methyleugenol. *Cell Death Dis* **2022**, *13*(11), 1009.

[6] Bolte, S.; Cordelières, F.P. A guided tour in subcellular colocalization analysis in light microscopy. *J. Microsc.* **2006**, *224(3)*, 213−232.
